# Leveraging UAV spectral and thermal traits for the genetic improvement of resistance to Dothistroma needle blight in *Pinus radiata* D.Don

**DOI:** 10.3389/fpls.2025.1574720

**Published:** 2025-06-17

**Authors:** Joane S. Elleouet, Russell Main, Robin J. L. Hartley, Michael S. Watt

**Affiliations:** ^1^ Data and Geospatial Intelligence, Scion, Wellington, New Zealand; ^2^ Data and Geospatial Intelligence, Scion, Rotorua, New Zealand; ^3^ Data and Geospatial Intelligence, Scion, Christchurch, New Zealand

**Keywords:** high-throughput phenotyping, needle disease resistance, hyperspectral imagery, thermal imagery, Dothistroma needle blight, *Pinus radiata*, single-step genomic evaluation

## Abstract

**Introduction:**

Phenotyping is critical in tree breeding, but traditional methods are often labour-intensive and not easily scalable. Resistance to biotic and abiotic stress is a key focus in tree breeding programmes. While heritable traits derived from spectral remote sensing have been identified in trees, their application to tree phenotyping remains unexplored. This study investigates *in-situ* high-throughput hyperspectral and thermal imaging for assessing Dothistroma needle blight (DNB) resistance in *Pinus radiata* D.Don.

**Methods:**

Using UAV-based hyperspectral and thermal imaging during a severe DNB outbreak in a clonal trial in New Zealand, we computed narrow-band hyperspectral indices (NBHIs), canopy temperature indices, radiative transfer inverted plant traits, and solar-induced fluorescence. Visual severity scores and remote sensing indices were modelled using spatially explicit mixed-effect linear models integrating pedigree and genomic data in a single-step genomic evaluation. Multi-trait models and sampling simulations were used to evaluate the potential of remote sensing indices to supplement or replace traditional phenotyping.

**Results:**

Remote sensing indices exhibited narrow-sense heritability values comparable to severity scores (up to 0.37) and high absolute correlation coefficients with severity scores (up to 0.79). Carotenoid and chlorophyll-related NBHIs were the most informative, reflecting physiological impacts of DNB. Combining partial visual scoring with NBHIs maintained high estimated breeding value (EBV) accuracy (0.68) at 50% scoring and moderate accuracy (0.59) at 20% scoring. EBV correlation with full scoring was above 0.8 even at 20% scoring. Using solely the most heritable NBHI achieved 0.71 breeding value accuracy and 0.79 absolute EBV correlation with severity scores, suggesting NBHIs can replace visual scoring with minimal precision loss.

**Discussion:**

By utilising UAV-based hyperspectral and thermal imaging to capture single-tree phenotypes related to disease in a forestry trial and pairing the data to genomic evaluation, this study establishes that remote sensing data offers an efficient, scalable alternative to traditional phenotyping. Our approach constitutes a major step towards characterising specific physiological responses, facilitating the discovery of the genetic architecture of physiological traits, and significantly enhancing genetic improvement.

## Introduction

Phenotyping refers to the process of observing and measuring an organism’s physical and biochemical traits. It is a fundamental step in the process of breeding to genetically improve species of commercial importance (Isik et al., 2017). It is however a time-consuming process, especially in large and long-generation organisms such as trees. Traditional tree phenotyping mostly relies on human assessment by foot in remote locations, and cannot easily be scaled up to larger trials, therefore limiting the amount of genetic material that can be tested. The precision of measurements is also limited for two main reasons. First, tree breeding trials are established in areas with limited control over environmental variables, creating extra variability in observable phenotypes. Secondly, many quantitative traits are measured subjectively using categorical scores with coarse scales, limiting the consistency of measurements within and across breeding trials, especially when the scoring is performed by multiple people (Poland and Nelson, 2011). The impacts of phenotyping limitations on selection efficacy and speed in tree breeding for forestry purposes have been referred to as the “phenotyping bottleneck” (Dungey et al., 2018). Remote sensing can alleviate many of these limitations by replacing or supplementing traditional measures of tree phenotypes, providing faster and objective measurements to ultimately improve genetic gains. High-throughput phenotyping has been in operation for more than a decade in common agricultural crop breeding systems (Song et al., 2021), with successful recent development of hyperspectral and thermal phenotyping using unmanned aerial vehicles (UAVs) in several crops (Liu et al., 2025; Zhang et al., 2025). However, its application in tree breeding still faces considerable challenges. Our research addresses the use of UAV hyperspectral and thermal measurements in breeding for disease resistance traits in a conifer species widely used in forestry, *Pinus radiata* D.Don (radiata pine).

Radiata pine is one of the most commercially important tree species in the southern hemisphere and the most planted forestry species in New Zealand. A breeding programme was established in New Zealand in the 1950s and remains very active. As with many pine species, radiata pine is prone to infection and damage by foliar diseases. Dothistroma needle blight (DNB), which is primarily caused by the pathogen *Dothistroma septosporum* (Dorog.) M. Morelet (Barnes et al., 2004), is characterised by the appearance of brick-red bands (1–3 mm wide) on needles, expanding to tissue dessication and eventually needle loss. The substantial productivity losses associated with the disease in Australia and New Zealand have prompted the inclusion of DNB resistance in radiata pine breeding programmes (Carson, 1989; Dungey et al., 2018). Screening for DNB symptoms is routinely performed in nurseries and breeding trials, and as DNB primarily affects juvenile stands (Bulman et al., 2013), the disease can occasionally occur in genetics trials before or at the usual selection age (6–9 years post establishment). Field phenotyping for resistance to DNB is undertaken using severity scores that are determined by a visual assessment of the percentage of the tree crown that is affected, in 5% increments (Bulman et al., 2004). Breeding for DNB resistance would therefore likely benefit from a more precise, objective and scalable phenotyping approach. Recent research on infected breeding trials reported narrow-sense heritability values for visual severity scores mostly between 0.29 and 0.35 (Li et al., 2018; Ismael et al., 2020; Klápště et al., 2020) but up to 0.43 (Klápště et al., 2020).

Remote sensing research has shown promising results in phenotyping individual tree health within forests and tree crops by detecting changes in physiological functioning caused by biotic stressors (Hernández-Clemente et al., 2019; Smigaj et al., 2023). Paired with UAV technology, remote sensing offers an efficient way to repeatedly collect high spectral and spatial resolution data (Smigaj et al., 2019; Oerke, 2020; Pan et al., 2023). Spores from *Dothistroma ssp.* infect pine needles through the stomata and create chlorotic lesions that expand into visible necrotic bands caused by the polyketide toxin, dothistromin (Bassett et al., 1970). Dothistromin targets chloroplasts and degrades photosynthetic pigments (Bradshaw, 2004; Kabir et al., 2015). Vegetation indices from spectral imagery can therefore detect DNB infection and characterise severity, as demonstrated in lodgepole pine (Smigaj et al., 2019) and radiata pine (Watt et al., 2023a, b). Water loss is commonly associated with foliar diseases, and a common physiological response is the closure of stomata to reduce transpiration. This leads to a reduction in stomatal conductance and assimilation rate, which can be detected with vegetation indices (Hernández-Clemente et al., 2019), and to an increase in leaf temperature, which can be detected using thermal sensing at the canopy level, as demonstrated in Scots pine (Smigaj et al., 2019). The use of UAV hyperspectral data has been extended to the combination of vegetation indices with plant functional traits inferred from radiative transfer modelling, improving disease severity prediction from the use of vegetation indices alone (Watt et al., 2023b).

The potential for spectral data and vegetation indices to be used as phenotypes in breeding has been explored in a number of tree species. Virlet et al. (2015) used multispectral imagery to identify quantitative trait loci associated with yield in an apple tree hybrid population and found broad-sense heritabilities between 0.60 and 0.77 for three multispectral indices. Tao et al. (2021) assessed the heritability of several UAV-derived vegetation indices to use in tree growth assessments in slash pine (*Pinus elliottii*) and found narrow-sense heritabilities ranging from 0.07 to 0.26. Čepl et al. (2018) conducted a similar study on Scots pine and found a similar range of heritabilities for vegetation indices (0.02 to 0.22) and up to 0.39 for single wavelength bands at the red edge inflection point, without however relating these metrics to any measured physiological trait. Corbin et al. (2024) used a handheld spectrometer to measure vegetation indices in common gardens of *Populus fremontii* and calculated broad-sense heritabilities up to 0.48. These exploratory studies showed that some spectral measurements can be heritable enough to be used in breeding. However, further research is needed to relate those measurements to specific adaptive or desirable traits in commercially planted tree species and to implement them within breeding trials in a large-scale phenotyping setup involving UAVs. To our knowledge, there has been no such attempt to date and no genetic assessment of spectral or thermal traits for selection of specific characteristics in tree breeding programmes. Here we address this key research gap by calculating tree-level UAV-collected hyperspectral and thermal measurements in a radiata pine breeding trial experiencing a disease outbreak and using derived indices to directly assess disease susceptibility for genetic selection.

Using UAV collected hyperspectral and thermal data, this study assesses how high-throughput remote sensing data can replace or complement visual severity scoring for DNB resistance. Remote sensing data was collected from a clonal breeding trial located in the central North Island of New Zealand at the peak of a DNB outbreak and linked with severity scores of the disease. We used narrow-band hyperspectral indices (NBHIs), thermal indices and plant traits calculated at the tree level as a means to supplement or replace time consuming measurements of disease severity. We first identify the most promising indices for use in phenotyping DNB severity for breeding. We then focus on two approaches for using the selected indices. The first approach assesses whether high-heritability indices can complement visual scoring, either by enhancing the accuracy of estimated breeding values (EBVs) for severity scores measured on all trees, or by maintaining high accuracy of breeding values for severity scores measured on a reduced proportion of the trial. The second approach determines whether using only remote sensing indices is a viable option to phenotype breeding trials for DNB resistance.

## Materials and methods

### Genetic material

**Figure 1:**
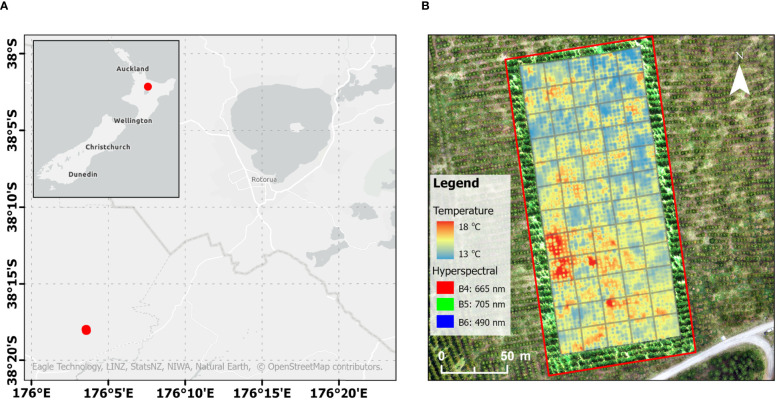
**(A)** Location of the trial site in the Central North Island, Rotorua, New Zealand. **(B)** Visualisation of the captured hyperspectral data (within the red box) overlaid with thermal data.

The study focuses on a clonally replicated breeding trial established by the New Zealand Radiata Pine Breeding Company (RPBC). The trial is in Kinleith forest in the Waikato region of New Zealand (**Figure 1A**) and comprises 60 6×6 row-column incomplete blocks of 36 single tree plots, with genotype allocation following the “Optimal Design” described by Butler (2013), which takes into account potential spatial correlations in rows and columns within blocks. In total, 1886 genotypes from 163 full-sib families (181 parents) are represented in the trial, with 1–3 ramets each. A pedigree with information for up to 5 generations was available for all tested genotypes. Genomic data was available for 65% of the tested genotypes and their parents, consisting in a set of ∼ 9.5k single nucleotide polymorphisms (SNPs) selected from two arrays: one from exome capture (Telfer et al., 2019) and the second one from a custom radiata pine Affymetrix Axiom array, NZPRAD02 (Graham et al., 2022). Markers in NZPRAD02 have a reasonably even distribution across linkage groups (Graham et al., 2022) and a set of markers almost identical to this used in this study has successfully been used for pedigree reconstruction in radiata pine (Klápště et al., 2022). Genotypes with <0.2 call rate and SNPs with <0.1 call rate were discarded from the analysis. The final genomic dataset had 1453 genotypes and 9570 markers.

### Disease assessments

A DNB outbreak was observed in the trial at year 5 after trial establishment. At the peak of the outbreak, severity scores were obtained on 25 September 2023 for all live trees in the trial (*n*= 2054). The severity was determined as the percentage of the crown affected by the disease, in 5% steps (Bulman et al., 2004). As the disease moves upwards from the base of the crown, this scoring system provides an objective means of characterising disease severity.

### Remote sensing captures

**Figure 2:**
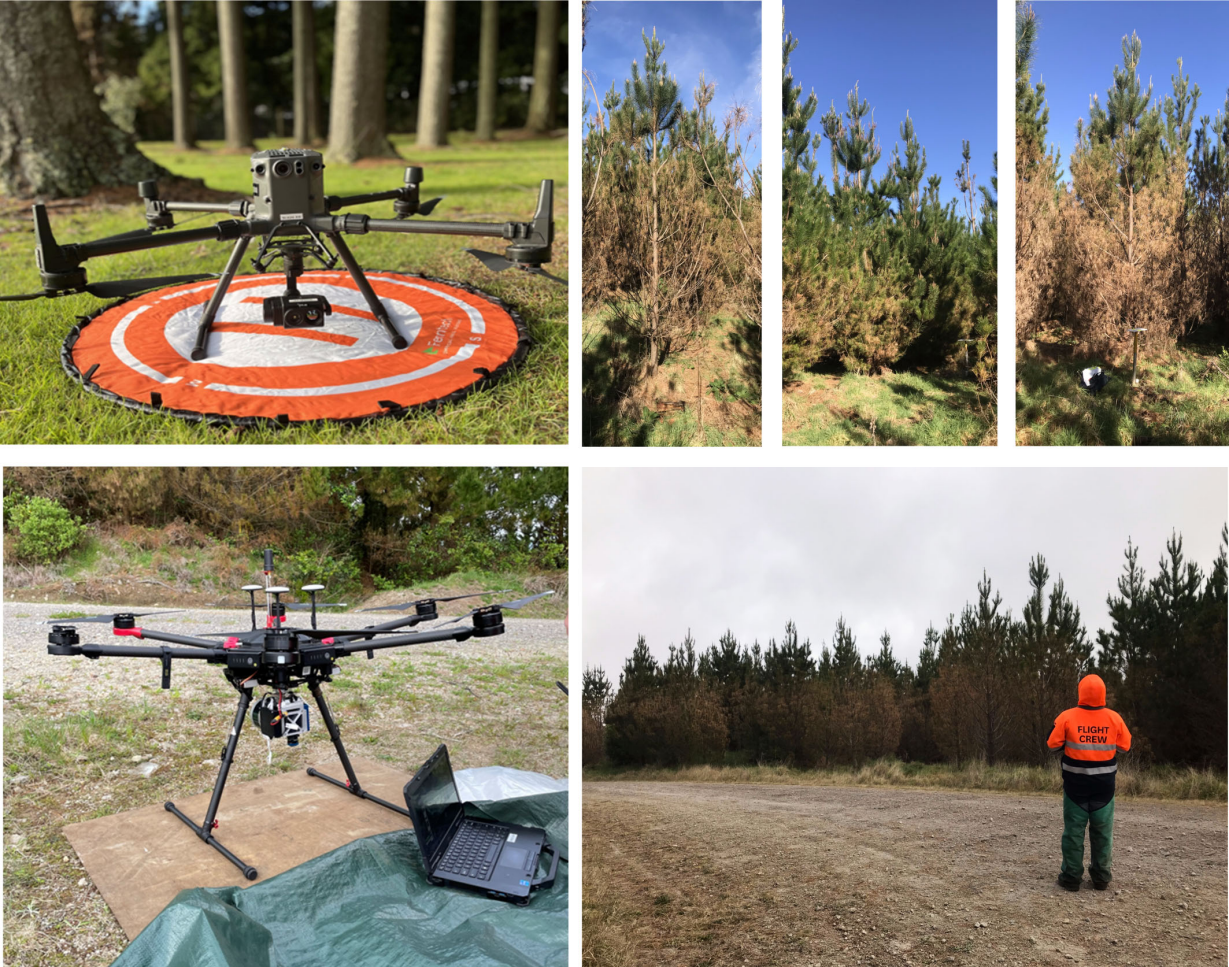
The UAV platforms used for data acquisition, namely the DJI Matrice 300 with FLIR Vue^®^ TZ20-R thermal sensor (top left) and DJI Matrice 600 with HeadWall Nano hyperspectral sensor (bottom left). Also shown are examples of trees exhibiting Dothistroma symptoms (top right) and a UAV pilot in the process of capturing data over the site (bottom right).

Remote sensing data captures were undertaken from a UAV close to the time of visual scoring (**Figures 1B**, **2**). Hyperspectral data were collected on 20 October 2023 using a Nano series HeadWall Photonics sensor (HeadWall Photonics, Inc., Bolton, MA, USA) mounted on a DJI Matrice 600 Pro platform (DJI, Shenzhen, China). The sensor featured a 17 mm focal length, with a 15° angular field of view (FOV). The collected hyperspectral imagers consisted of 270 spectral bands across the visible to near-infrared (VNIR) range from 400–1000 nm with a 6 nm full width at half maximum (FWHM). The UAV was flown at mid-day at 6 m/s and 120 m altitude with 40% side overlap, resulting in a 5 cm/pixel ground sampling distance (GSD). Solar irradiance measurements across a range of 350–2500 nm were recorded concurrently every 15 seconds using a Spectral Evolution RS-5400 spectroradiometer (Spectral Evolution, Haverhill, MA, USA). The raw digital numbers from the hyperspectral data were processed to orthorectified reflectance cubes using SpectralViewTM software (Headwall Photonics, Fitchburg, MA, USA). Radiance was calculated with dark current corrections and calibration files, then converted to reflectance using a 3 × 3 m² ground calibration target located within the flightpath. The reflectance data cubes underwent an orthorectification process to accurately geolocate the imagery and correct any roll, pitch, and yaw imagery distortions. Using post-processed RTK files, previously collected high-resolution digital terrain models (DTM), RGB ortho-mosaics, and the GCPs, the reflectance cubes were orthorectified, mosaiced, and registered to the Universal Transverse Mercator (UTM), Zone 60 South, projection.

Thermal data were collected on 13 October 2023 using a FLIR Vue^®^ TZ20-R sensor (Teledyne FLIR, USA) mounted on a DJI Matrice 300 RTK platform (DJI, Shenzhen, China). The sensor has a 12 μm pixel pitch, operates within the 7.5–13.5 μm spectral range, and has a reported thermal sensitivity of 85 mK (at F/1.0). To support flight parameterisation and thermal index calculations, ambient air temperature and relative humidity were continuously recorded using an HMP155A probe (Vaisala, Vantaa, Finland) connected to a CR1000 datalogger (Campbell Scientific, Logan, UT, USA). Prior to data acquisition the thermal camera was powered on and allowed to stabilise for 15 minutes. Ambient temperature, relative humidity, and an emissivity value of 0.98 were entered into the DJI Pilot controller software (DJI, Shenzhen, China). Data were captured using the 4.9 mm lens (95°FOV) at an altitude of 80 m and a flight speed of 3 m/s, with 90% forward and side image overlap. The resulting imagery had an approximate ground resolution of 23 cm, with radiometric temperature values embedded in each pixel and stored in the proprietary R-JPEG format. Images were subsequently processed and orthomosaicked in Pix4D using the FLIR Vue Pro R camera profile.

To enable extraction of pure canopy pixels from thermal and hyperspectral data, LiDAR was collected and processed following Watt et al. (2024b). This facilitated individual tree segmentation and the generation of canopy polygons for guiding image-based data extraction. LiDAR was acquired on 8 September 2023 using the DJI Zenmuse L1 sensor on the Matrice 300 RTK platform (DJI, Shenzhen, China), flown at 55 m altitude and 3 m/s in a grid pattern with 10 m line spacing, achieving 85% overlap. The system operated in repetitive scan mode at 160 Hz, recording up to three returns per pulse. For accurate geolocation, the DJI Matrice 300 used a real-time kinematic (RTK) base station and 11 high-contrast ground control points (GCPs) with matte black and retro-reflective white panels, evenly distributed across the site. GCP centers were identified from the LiDAR point cloud and used to georeference the imagery. Post-capture, standard pre-processing steps were undertaken that included denoising, ground point classification, digital terrain model (DTM) generation and ground normalisation using LASTools (v2.0.2) following (Watt et al., 2024b).

Tree detection and crown delineation were performed in R (R Core Team, 2023). A 25 cm resolution canopy height model (CHM) was generated using the pit-free method of (Khosravipour et al., 2014) and then smoothed with a 3×3 pixel moving window. Tree peaks were identified using a local maxima algorithm with a 3 m window, reflecting initial planting spacing, and verified against high-resolution imagery and field-validated stem maps. The “mcws” function in the ForestTools R package (Plowright, 2025) was used to delineate crowns via the watershed algorithm, restricting delineation to the top 75% of the CHM to avoid low vegetation. The resulting crown polygons were visually checked and erroneous crowns removed.

### Extraction of hyperspectral and thermal predictors

The hyperspectral and thermal data extracted for each canopy was used to calculate several plant traits and indices that could highlight tree sensitivity to infection. A range of narrowband hyperspectral indices (NBHI), designed to capture variations in plant biochemical and structural properties, were calculated using the formulations in **Supplementary Table S1**.

The PRO4SAIL2 radiative transfer model, which couples the leaf model PROSPECT-D (Féret et al., 2017) with the canopy model 4SAIL2 (Verhoef and Bach, 2007), was used to simulate canopy structural traits and leaf biochemical properties from pure vegetation pixels in the radiata pine canopies. By isolating pure canopy pixels, the influence of shade and soil was minimised reducing structural artifacts (Zarco-Tejada et al., 2021). This enabled accurate simulation of chlorophyll a+b (C_a+b)_, anthocyanin (Anth.), and carotenoid (C_x+c_) concentrations as well as structural parameters such as the leaf inclination distribution function (LIDF_a_) and leaf area index (LAI). A lookup table with 200,000 simulations was generated in forward mode by running the PRO4SAIL2 model, using the parameters and ranges in **Supplementary Table S2** which were randomly varied based on continuous uniform distributions, with other model settings kept at default. Data resampling was undertaken to match the hyperspectral sensor’s spectral resolution using a Gaussian response function. Model inversion was performed using Support Vector Regression (SVR), a non-parametric approach grounded in statistical learning theory (Vapnik, 1999). SVR models were trained in parallel using MATLAB (Statistics and Machine Learning, Parallel Computing, and Deep Learning toolboxes; MathWorks, Natick, MA, USA), with resampled reflectance as input and predicted biochemical and structural traits as outputs. A radial basis function kernel was used, with hyperparameters optimised during training.

Solar-induced fluorescence (SIF), a proxy for photosynthetic efficiency, was estimated using hyperspectral radiance and solar irradiance data through the Fraunhofer Line Discriminator (FLD) approach (Plascyk, 1975). Specifically, the three-band FLD method (3FLD) proposed by Maier et al. (2004) was applied. This method extends the traditional FLD technique by incorporating three spectral bands — two situated outside the O_2_-A absorption band and one within it — to improve the accuracy of SIF retrievals.

Thermal imagery data and field-measured ambient temperature were used to compute the normalised canopy temperature index (T_c_ – T_a_), where T_c_ represents canopy-level temperatures and T_a_ reflects the ambient air temperature recorded during the flight period (Smigaj et al., 2023).

### Univariate analyses of severity scores and remote sensing traits

Single phenotypes from visual assessment (severity scores) and remote sensing captures were analysed using the following linear mixed model implemented in the ASReml-R statistical package (Butler et al., 2009):


$$\text{y}=\text{X}b+{\text{Z}}_{\text{a}}{\text{u}}_{\text{a}}+{\text{Z}}_{\text{g}}{\text{u}}_{\text{g}}+{\text{Z}}_{\text{d}}{\text{u}}_{\text{d}}+e$$

where *y* is the vector of observed single-tree phenotypes, *b* is the vector of fixed effects with the associated design matrix ***X***, $u_{a}$ , $u_{g}$ , and $u_{d}$ are the vectors of random additive genetic, nonadditive genetic, and design effects, respectively, with associated design matrices $Z_{a}$ , $Z_{g}$ , and $Z_{d}$ , and ***e*** is the vector of residuals. Random effects are normally distributed following ${\text{u}}_{\text{a}}∼\;N(0,\text{H}\sigma_{a}^{2})$ , ${\text{u}}_{\text{g}}\;∼\;N(0,\text{I}\sigma_{g}^{2})$ , and ${\text{u}}_{\text{d}}\;∼\;N(0,\text{I}\sigma_{d}^{2})$ , where ***I*** is the identity matrix, ***H*** is the average numerator relationship matrix between genotypes, and $\sigma_{a}^{2}$ , $\sigma_{g}^{2}$ , and $\sigma_{d}^{2}$ are variances for additive genetic, nonadditive genetic, and design effects, respectively. In this model, which is hereafter referred to as non-spatial, residuals are independent, following $\text{e}\;∼\;N(0,\text{I}\sigma_{e}^{2})$ . To account for the spatial correlation between trees in the trials, we consider an alternative model which allocates a separable first-order autoregressive process component to residuals. In this case, e ∼ N(0,R) where $\text{R}=[AR1(\rho_{col})⊗AR1(\rho_{row})]\sigma_{\xi }^{2}+\text{I}\sigma_{\eta }^{2}$ . AR1(ρ) is a first-order autoregressive correlation matrix with autocorrelation parameter ρ , and $\sigma_{\xi }^{2}$ and $\sigma_{\eta }^{2}$ are spatial and independent residual variances, respectively.

In the first model instance, the average numerator relationship matrix ***H*** is the pedigree-based relationship matrix ***A*** calculated from the pedigree on all genotypes present in the trial. Alternatively, a blended matrix $H_{\lambda }$ was used for the variance structure of the additive genetic effects. $H_{\lambda }$ was computed for all genotypes using ***A*** and the genomic marker-based relationship matrix ***G***, calculated on genotyped individuals. ***A*** and ***G*** were calculated using the AGHmatrix R package (Amadeu et al., 2016), using the VanRaden method for ***G*** (VanRaden, 2008). The blended genetic relationship matrix $H_{\lambda }$ was calculated using the ASRgenomics package (Gezan et al., 2022) and can be defined by its inverse as follows:


$$H_{\lambda }-1=A^{-1}+[\begin{array}{cc} 0 & 0\\ 0 & \lambda ({\text{G}}^{-1}-{\text{A}}_{22}-1) \end{array}]$$

where $A_{22}$ is the genetic relationship matrix for genotyped individuals and *λ* is a scaling factor bound between 0 and 1 representing the weight associated to ***G*** relative to ***A*** for genotyped individuals.

Thorough model building and model selection were performed on severity scores. The Bayesian Information Criterion (BIC) as well as graphical checks of model diagnostics and variograms were used to guide model selection. One-sided likelihood ratio tests on random effect variances were used to compare models with nested random effects, and incremental addition or removal of fixed effects was guided by the Wald test. The model was constructed using the pedigree-based relationship matrix ***A***. We started with a base model with an intercept, additive ( $u_{a}$ ) and nonadditive ( $u_{g}$ ) genetic effects, and block as a design effect ( $u_{d}$ ). Cubic spline transformations of trial rows and columns were added as fixed effects in ***b*** after visual examination of residuals against trial rows and columns, to account for environmental effects at the trial level. Two alternative spatial models for residuals were tested, one assuming no independent residual component ( $\sigma_{\eta }^{2}=0$ ), and one including the residual component. Once the most appropriate residual structure was selected, a reassessment of the relevance of block effects and row and column spline effects was conducted.

As part of the analysis of severity scores, after selecting the final set of fixed and random effects and the appropriate residual structure, we fitted the model using $H_{\lambda }$ and tested different values of *λ* in 0.1 increments across its possible range. The optimal *λ* value was selected based on model BIC. The final model including the optimal $H_{\lambda }$ matrix was implemented for the univariate genetic analyses of all remote sensing traits.

Narrow-sense heritability was calculated as follows:


$$\hat{h^{2}}=\frac{\hat{\sigma_{a}^{2}}}{\hat{\sigma_{a}^{2}}+\hat{\sigma_{g}^{2}}+\hat{\sigma_{r}^{2}}}$$

where $\hat{\sigma_{r}^{2}}=\hat{\sigma_{e}^{2}}$ for non-spatial models and $\hat{\sigma_{r}^{2}}=\hat{\sigma_{\eta }^{2}}$ for spatial models.

Estimated breeding values were extracted from random genotype effects and their accuracy was obtained from univariate models using the following calculation:


$$accuracy_{i}=\;\sqrt{1-\frac{\hat{SE_{i}2}}{\hat{\sigma_{a}^{2}}}}$$

where $SE_{i}$ is the estimated standard error of the breeding value for genotype *i*.

### Multi-trait analyses

Multi-trait analyses consist of simultaneously modelling several response traits and allow estimating the correlations between traits through structured variance matrices. In our case, the genetic correlation is estimated through appropriate use of the genetic relationship matrix. When the genetic correlation between two traits is non-zero, prediction performance of genotypes for one or more of the modelled traits can be improved. In our case, multi-trait analyses are advantageous when the trait in focus for selection has a lower heritability than other measured correlated traits. They can also be used when measurements for the focus trait are only available for a subset of observations in the trial: the trait can still be predicted from unmeasured units using the correlation with other traits that have been measured. The general multivariate model with *d* traits is as follows:


$$\text{y}=\text{X}b+{\text{Z}}_{a}{\text{u}}_{a}+{\text{Z}}_{g}{\text{u}}_{\text{g}}+{\text{e}}_{\xi }+{\text{e}}_{\eta }$$

where $\text{y}={\left[{\text{y}}_{1},\ldots ,{\text{y}}_{\text{d}}\right]}^{T}$ is the stacked column vector of phenotypic observations relating to each trait. Secondary traits $y_{2},\ldots ,y_{d}$ were rescaled to a similar scale as $y_{1}$ if necessary, to improve convergence of the restricted maximum likelihood (REML) algorithm. $\text{b}={\left[{\text{b}}_{1},\ldots ,{\text{b}}_{\text{d}}\right]}^{T}$ is the column vector of fixed effects selected in univariate analyses (intercept and spline functions of trial rows and columns) with the associated design matrix $\text{X}=[\begin{array}{ccc} {\text{X}}_{1} & \cdots & 0\\ \vdots & \ddots & \vdots \\ 0 & \cdots & {\text{X}}_{\text{d}} \end{array}]$ . ${\text{u}}_{\text{a}}={\left[{\text{u}}_{\text{a}1},\ldots ,{\text{u}}_{\text{a}d}\right]}^{T}$ and ${\text{u}}_{\text{g}}={\left[{\text{u}}_{\text{g}1},\ldots ,{\text{u}}_{\text{g}d}\right]}^{T}$ are the column vectors of random additive genetic and nonadditive genetic effects, respectively, with associated design matrices ${\text{Z}}_{a}=[\begin{array}{ccc} {\text{Z}}_{\text{a}1} & \cdots & 0\\ \vdots & \ddots & \vdots \\ 0 & \cdots & {\text{Z}}_{\text{a}d} \end{array}]$ and ${\text{Z}}_{\text{g}}=[\begin{array}{ccc} {\text{Z}}_{\text{g}1} & \cdots & 0\\ \vdots & \ddots & \vdots \\ 0 & \cdots & {\text{Z}}_{\text{g}d} \end{array}]$ . In this model notation we separated the residuals into a spatially structured component $e_{\xi }$ and an independent component $e_{\eta }$ , where ${\text{e}}_{\xi }={\left[{\text{e}}_{\xi 1},\ldots ,{\text{e}}_{\xi \text{d}}\right]}^{T}$ and ${\text{e}}_{\eta }={\left[{\text{e}}_{\eta 1},\ldots ,{\text{e}}_{\eta \text{d}}\right]}^{T}$ . Random effects and residuals are modelled as follows: $u_{g}\;∼\;N(0,\sum_{g}\;⊗I_{p})$ , $u_{a}\;∼\;N(0,\Sigma_{a}\;⊗{\text{H}}_{\text{λ}})$ , ${\text{e}}_{\xi }\;∼\;N(0,\;\Sigma_{\xi }⊗{\text{R}}_{\text{ξ}})$ , and $e_{\eta }\;∼\;N(0,{\text{Σ}}_{\text{η}}⊗I_{n})$ , where $\Sigma_{g}$ , $\Sigma_{a}$ , $\Sigma_{\xi }$ , and ${\text{Σ}}_{\text{η}}$ are general heterogenous variance structures of the form


$$\sum_{\text{x}}=[\begin{array}{ccc} \sigma_{x}{\;}_{1}^{2} & \cdots & \rho_{x_{1d}}\sigma_{x_{1}}\sigma_{x_{d}}\\ \vdots & \ddots & \vdots \\ \rho_{x_{d1}}\sigma_{x_{1}}\sigma_{x_{d}} & \cdots & \sigma_{x}{\;}_{d}^{2} \end{array}]$$

$I_{n}$ and $I_{p}$ are identity matrices of sizes *n*, the number of trees in the trial, and *p*, the number of genotypes represented in the trial. $H_{\lambda }$ is the blended genetic relationship matrix. Finally, ${\text{R}}_{\text{ξ}}=[AR1(\rho_{col})⊗AR1(\rho_{row})]$ is the separable autoregressive variance structure of spatially correlated residuals. We note that this multivariate model has the same fixed effects, random effects, genetic relationship matrix and type of residual structure as the final univariate model selected for all studied traits. We also note that this model assumes the same spatial row and column autocorrelation values $\rho_{col}$ and $\rho_{row}$ across jointly modelled traits.

We first identified the remote sensing index with the highest observed correlation with severity scores. To estimate genetic correlations between the index and severity scores, and to assess whether joint modelling enhances the accuracy of estimated breeding values (EBVs) of severity scores, we performed a bivariate analysis with severity scores as the primary trait and the selected index as the secondary trait.

To test whether visual phenotyping can be reduced to a sample of trees in the trial and complemented by remote sensing data, we repeated and modified the previous bivariate analysis. Instead of using the complete set of severity scores as the primary trait, we randomly selected from 5% to 100% of severity scores and used the selected subset of severity scores as the primary trait. We repeated the procedure 10 times for each sampled proportion.

We then explored the use of remote sensing indices only in DNB severity phenotyping, by incrementally building a multi-trait model with a combination of high-heritability indices that are correlated with severity scores, starting with the most correlated index and adding additional indices in decreasing order of heritability values.

## Results

### Distribution and genetic analysis of severity scores

**Figure 3:**
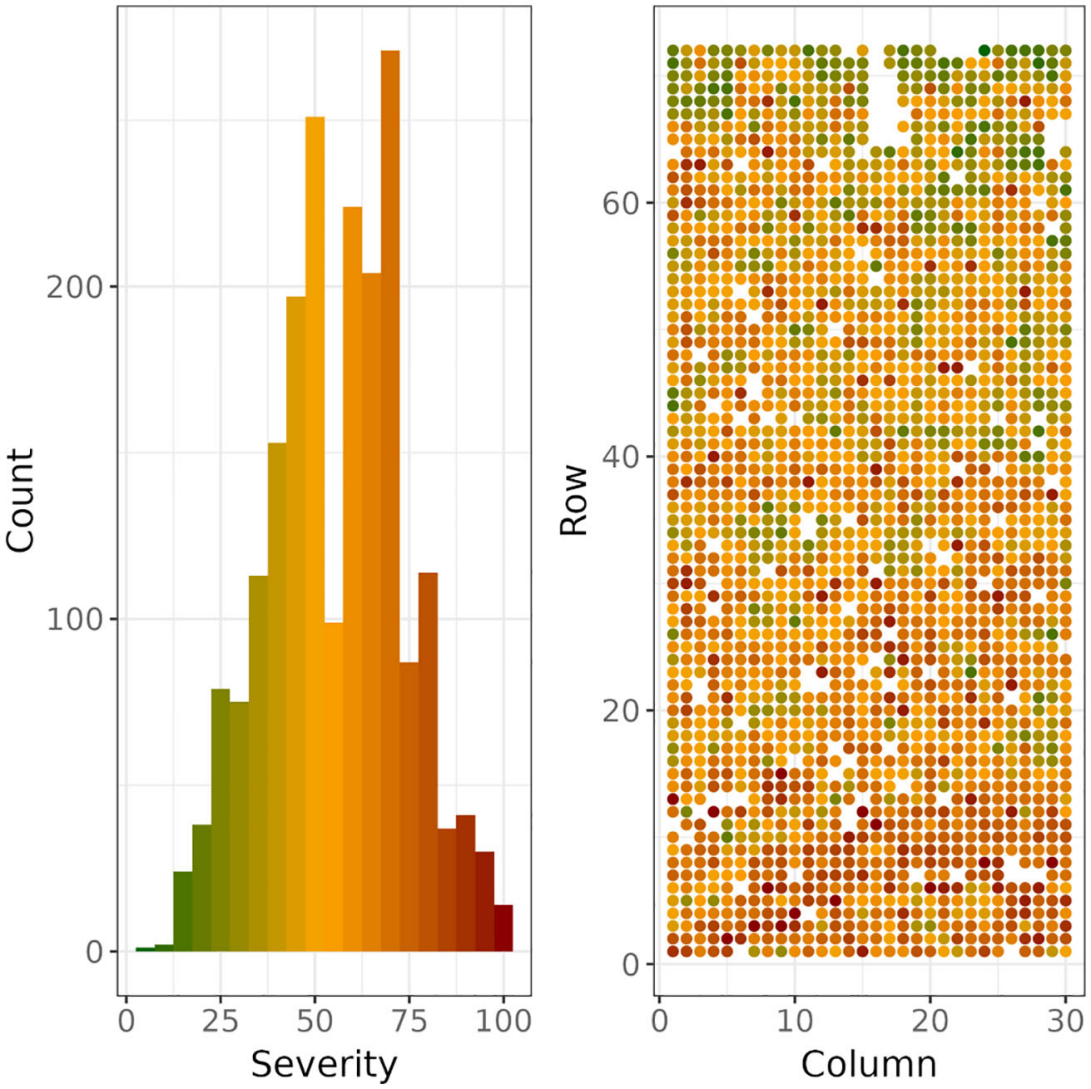
Histogram (left) of severity scores and their spatial distribution (right) across rows and columns in the trial. The coloured histogram (left) also acts as a colour legend for the spatial distribution of severity scores (right).

The distribution of DNB severity over the 2054 scored trees covered the full range of possible values, from 5% to 100%, with a median score of 55% (**Figure 3**). Tree-level and trial-level spatial dependence is apparent, (**Figure 3**), suggesting that tree-to-tree transmission of the disease and environmental heterogeneity in the trial had an influence on disease severity.

Selected genetic models of severity scores were in accordance with this observation. Model selection was performed using the pedigree-based relationship matrix *A* for the estimation of additive genetic effects. Including a spatial autocorrelation variance structure of residuals led to a significantly better fit than modelling independent residuals (**Table 1**), and the addition of splines for trial rows and columns in the fixed effects also improved the model. These results suggest both a landscape effect (variation across the trial site) and a neighbour effect (proximity related disease transmission) on the severity of disease. The addition of a spatially independent residual term gave a likelihood ratio test p-value of 0.05. Row and column correlations were around 0.5 and the spatially dependent error variance was five times higher than the independent error variance, suggesting that most of the residual structure could be accounted for by spatial factors. However, as the best model overall (lowest BIC) included both separable autoregressive and spatially independent error terms, both were included in the final model. As block effects became insignificant after accounting for spatial structure in the residuals, we omitted the block term from the final model, effectively pooling any block effect variance with the residual variance. **Table 1** shows variance components and estimated genetic parameters for the best spatial and non-spatial models when using the ***A*** matrix.

**Table 1:** Parameter estimates, narrow-sense heritability, Bayesian information criterion (BIC) and estimated breeding value (EBV) accuracy for four alternative models of Dothistroma needle blight severity scores.

Model type	Non-spatial	Spatial	Non-spatial	Spatial
Genetic relationship matrix	A	A	H_0.5_	H_0.5_
Model statistics				
BIC	12659.52	12455.74	12650.55	12448.6
Variance components				
Additive genetic variance	63.37 (12.60)	61.13 (11.79)	89.83 (16.82)	79.03 (14.19)
Nonadditive genetic variance	37.96 (12.66)	36.9 (10.59)	27.14 (16.82)	31.41 (10.73)
Independent residual variance	117.46 (10.08)	25.43 (11.5)	117.70 (10.08)	27.83 (11.3)
Design (block) variance	21.37 (5.78)		20.95 (5.66)	
Spatial residual variance		115.8 (11.63)		112.87 (11.36)
Column AR1 correlation		0.49 (0.05)		0.5 (0.05)
Row AR1 correlation		0.52 (0.05)		0.52 (0.05)
Genetic parameters + predictions				
Narrow-sense heritability	0.29 (0.05)	0.50 (0.08)	0.38 (0.06)	0.57 (0.08)
Mean EBV accuracy (all)	0.66 (0.00)	0.67 (0.00)	0.76 (0.00)	0.74 (0.00)
Mean EBV accuracy (clones)	0.70 (0.00)	0.72 (0.00)	0.79 (0.00)	0.77 (0.00)
Mean EBV accuracy (ancestors)	0.50 (0.01)	0.52 (0.01)	0.64 (0.01)	0.61 (0.01)

Numbers in brackets are standard errors.

The test on proportion of ***G*** vs. ***A*** matrices in the blended $H_{\lambda }$ matrix was performed using the model selected above. We found that values of *λ* between 0.4 and 0.6 were optimal. We set λ=0.5 as it provided both the lowest BIC and highest heritability for severity scores. The final model selected to calculate and compare breeding values across traditional and remote sensing traits was therefore the spatial model with the $H_{0.5}$ matrix, and its variance components and estimated genetic parameters are presented in **Table 1**, together with results for the best non-spatial model for comparison purposes. Narrow-sense heritability of severity scores using $H_{0.5}$ was 0.38 with a standard error (SE) of 0.06 when estimated from the non-spatial model and 0.57 (SE=0.08) when estimated from the final spatial model.

### Univariate analysis of remote sensing indices

**Figure 4:**
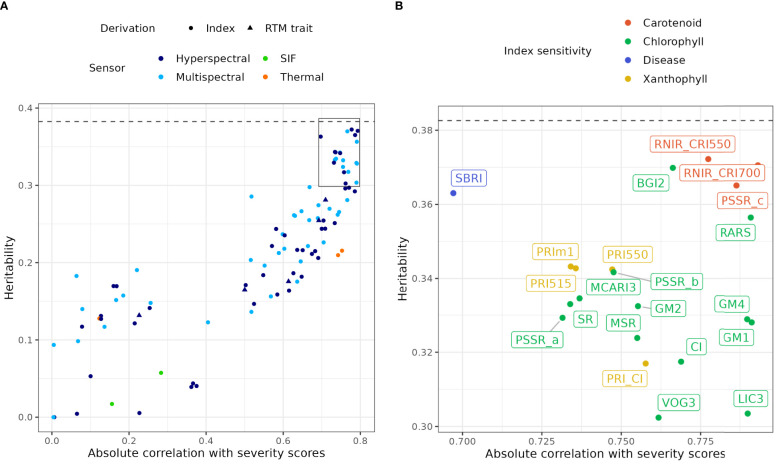
**(A)** Relationship between narrow-sense heritability estimated from univariate models of all remote sensing indices, and their absolute observed correlation with severity scores, by sensor type and derivation type. Note that the multispectral category corresponds to hyperspectral indices whose calculation is compatible with multispectral capture. The dashed line indicates the estimated narrow-sense heritability of severity scores. The box delineates the area of **(B)**. RTM, Radiative transfer model; SIF, Solar-induced fluorescence. **(B)** Expansion of **(A)** for indices with heritability above 0.3, with point and index labels coloured by sensitivity to different physiological characteristics.

The same final univariate spatial model and $H_{0.5}$ matrix that were used in the analysis of visual severity scores were also applied to model the genetic parameters of each remote sensing metric. **Supplementary Table S1** reports narrow-sense heritability for each remote sensing index, estimated from variance components from non-spatial models with design effects. Calculating heritability on the non-spatial model with design effects makes values more comparable across indices as design effects are the same across indices and all the residual variance is included in the calculation. Narrow-sense heritability of remote sensing indices ranged from 0 to 0.37 when estimated from non-spatial models, reaching values similar to severity scores (0.38). Absolute observed correlations with severity scores reached 0.79 (**Supplementary Table S1**). The relationship between heritability (h^2^) and absolute Pearson’s correlation with severity scores (r_P_) pictured in **Figure 4A** allows the identification of indices with high heritability and high absolute correlation with severity scores. These traits are potential candidates to assist or replace visual scoring in genetic selection for DNB resistance.

The most promising indices were mostly NBHIs (**Figure 4B**), some of which included spectral bands that can be calculated from multispectral instruments (**Supplementary Table S1**). The index RNIR_CRI700, RNIR_CRI550 and PSSR_c were the best performing indices (**Figure 4B**, **Supplementary Table S1**), which are proxies for variation in carotenoid content: RNIR_CRI550 and RNIR_CRI700 (h^2^ = 0.37), are carotenoid reflectance indices (Gitelson et al., 2003, 2006). RNIR_CRI700 is also the NBHI with the highest absolute correlation with severity scores (r_P_=-0.79). Many other important indices (CI, GM1, GM2, GM4, PSSR_a, PSSR_b, VOG3, BGI2, LIC3) characterise chlorophyll content and three were sensitive to both chlorophyll content and plant structure (MCARI3, MSR, SR). Notably, none of the water indices had either high heritability (h^2^ = 0.004 - 0.04) or high correlation with disease (0.065 - 0.375). Mean and median normalised canopy temperature (TcTa_InMn and TcTa_InMed), derived from thermal captures, were highly correlated with severity scores but had a lower heritability. Solar-induced fluorescence only had a low to moderate correlation with severity scores and low heritability. Traits inverted from radiative transfer modelling were not as useful as the best NBHIs and mainly had heritability values between 0.15 and 0.28.

**Figure 5:**
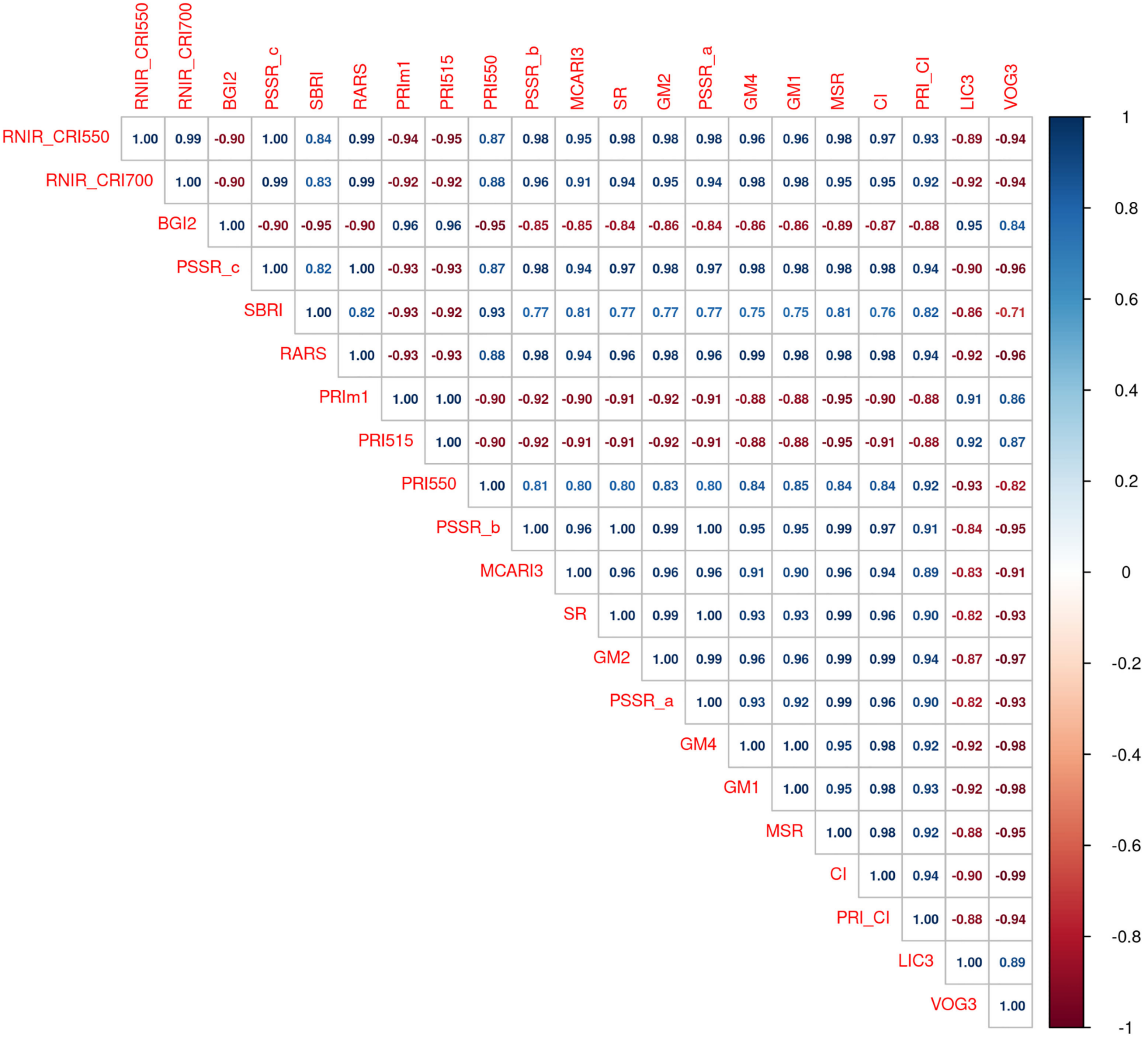
Pairwise correlation between indices with heritability values above 0.30. The left-to-right and top-to-bottom order of indices reflects their ranking in decreasing heritability values.

We selected the 21 indices with heritability values above 0.30 (**Figure 4B**) and computed their pairwise correlations using Pearson’s correlation coefficient (**Figure 5**). Pairwise correlations ranged from 0.71 to 1. Many of these NBHIs were highly correlated to each other as they are derived from similar wavelengths (**Supplementary Table S1**). For instance, RNIR_CRI550 and RNIR_CRI700 (h^2^ = 0.37), are carotenoid reflectance indices (Gitelson et al., 2003, 2006) and were strongly correlated with each other (r_P_ > 0.99). RNIR_CRI700 is also the NBHI with the highest absolute correlation with severity scores (r_P_=-0.79). Most other indices with heritability values > 0.30 were strongly correlated with RNIR_CRI700, with Pearson’s correlation coefficients ranging from 0.83 to 0.99.

**Figure 6:**
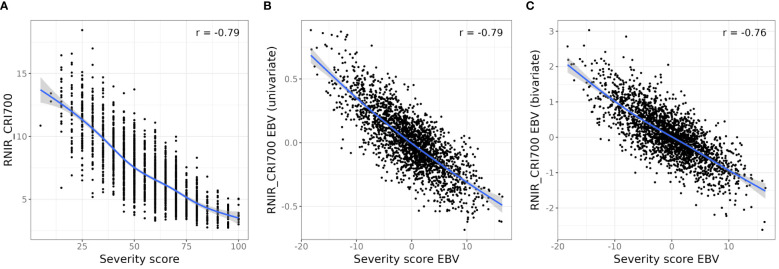
Relationship between severity scores and RNIR_CRI700. **(A)**. Relationship between RNIR_CRI700 and observed severity. **(B)** Relationship between RNIR_CRI700 estimated breeding values (EBVs) and EBVs from the univariate model. **(C)** Relationship between RNIR_CRI700 EBVs from the bivariate model with SIPI and severity EBVs from the univariate model. A smoothing spline (blue line) has been fitted to aid visualisation and Pearson’s correlation coefficient is shown for all relationships.

**Figure 6** shows the relationship between severity scores and RNIR_CRI700 both in terms of phenotypic observations (A) and EBVs from final spatial univariate models (B). Other promising indices included three Red/Green/Blue indices: the blue/green index (BGI2), the ratio analysis of reflectance spectra (RARS) and the Lichtenthaler index (LIC3). These indices use spectral bands compatible with multispectral capture. One high-heritability NBHI, the Sugar Beet Rust Index (SBRI), was developed for disease assessment (Mahlein et al., 2013). All other high-ranking indices are NBHI relating to pigments or structural characteristics, and their description can be found in **Supplementary Table S1**.

### Multi-trait analysis of severity scores

We performed a bivariate spatial analysis with the full set of severity scores as the primary trait and RNIR_CRI700 as the secondary trait, as its absolute correlation with severity scores was the highest (r_P_ = -0.79), and its relationship with severity scores appeared to be relatively linear (**Figure 6A**). The bivariate model estimated a genetic correlation of -0.80 (SE 0.09) between severity scores and RNIR_CRI700. The accuracy of severity score EBVs was on average 0.75. This represents little improvement from the mean accuracy of 0.74 obtained from the univariate analysis of severity scores, which is not surprising as severity scores have a higher estimated heritability than remote sensing indices.

**Figure 7:**
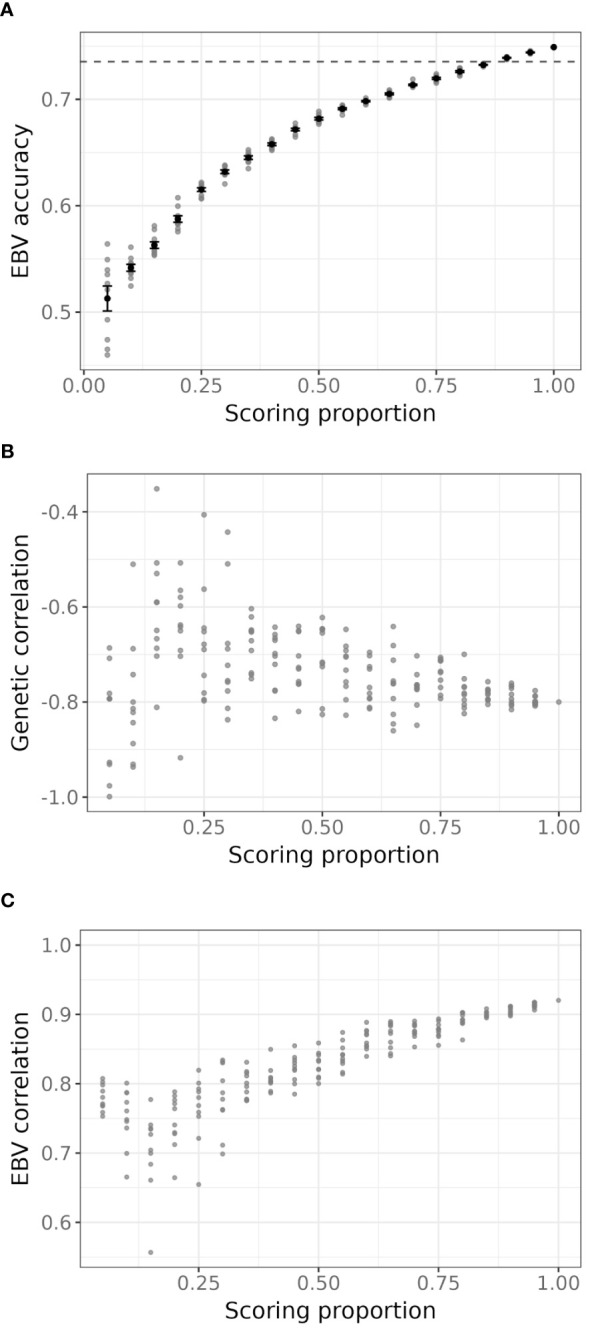
**(A)** Accuracy of estimated breeding values (EBVs) for severity scores for each proportion of severity scores sampled. Grey points represent each iteration, black points and error bars are the average and standard deviation for all 10 iterations of a given scoring proportion. The dashed line is the accuracy of severity scores EBVs from the univariate model using the full set of severity scores. **(B)** Estimated additive genetic correlation between severity scores and RNIR_CRI700 for each iteration of the simulation. **(C)** Pearson’s correlation coefficient between EBVs of sampled severity scores in each iteration of the bivariate model and EBVs from the univariate model using the full set of severity scores.

Results from all instances of the bivariate model with various proportions of scored trees as the primary trait and RNIR_CRI700 as the secondary trait are shown in **Figure 7**. When 80% or more trees are scored, mean EBV accuracy of severity scores were similar or lower than the EBV accuracy of severity scores from the univariate model, and steadily decreased to around 0.5 with 5% trees scored (**Figure 7A**). The precision and accuracy of genetic correlation estimates decreases as fewer trees are scored, with estimated values becoming unreliable below 40% of sampled trees (**Figure 7B**). Severity score EBVs are highly correlated to EBVs from the univariate model with full scoring, even at low scoring proportions (**Figure 7C**). However, a sharper decrease in correlation is observed when fewer than 25% trees are scored, with correlation values dropping to below 0.85.

### Multi-trait analysis of remote sensing indices

To assess the ability of remote sensing indices to fully replace severity scores in DNB resistance phenotyping, we selected the index most correlated with severity scores, RNIR_CRI700, as the primary trait in the spatial multi-trait analysis of remote sensing metrics. We selected remote sensing indices with estimated heritability higher than 0.2 and an absolute correlation with RNIR_CRI700 lower or equal to 0.90 as potential secondary traits. We avoided selecting indices with a higher absolute correlation as they would add little information compared to the univariate model of the primary trait and lead to multi-trait model convergence issues. Resulting EBV accuracies and EBV correlation with severity scores from a univariate model are presented in **Table 2**.

**Table 2:** Observed correlation, estimated genetic correlation, mean estimated breeding value accuracy and correlation with severity scores resulting from bivariate models with RNIR_CRI700 as the primary trait.

Trait2	Observed correlation	Genetic correlation	Mean RNIR_CRI700 EBV accuracy	Mean trait 2 EBV accuracy	RNIR_CRI700 EBV correlation with severity scores	Trait 2 EBV correlation with severity scores
SIPI	-0.78	-0.97	0.76	0.76	-0.76	0.76
BGI2	-0.90	-0.97	0.74	0.78	-0.55	0.48
SBRI	0.83	0.90	0.68	0.76	-0.60	-0.43
PRI550	0.88	0.86	0.65	0.76	-0.69	-0.47
MTVI2	0.84	0.88	0.62	0.70	-0.73	-0.55
OSAVI	0.86	0.86	0.62	0.71	-0.75	-0.54
R	0.87	0.84	0.61	0.72	-0.68	-0.39
RDVI	0.81	0.86	0.61	0.69	-0.75	-0.58
MSAVI	0.79	0.86	0.61	0.69	-0.75	-0.59
Car_RTM	0.84	0.85	0.60	0.68	-0.74	-0.53
LIC1	0.90	0.81	0.60	0.72	-0.77	-0.45
MTVI1	0.71	0.84	0.59	0.66	-0.76	-0.61
TVI	0.69	0.81	0.58	0.66	-0.77	-0.60
CAR	-0.88	-0.77	0.58	0.71	-0.78	0.46
PRIm4	0.82	0.73	0.58	0.72	-0.76	-0.42
GI	0.82	0.71	0.56	0.70	-0.76	-0.43
CLS	-0.63	-0.70	0.56	0.67	-0.78	0.51
G	0.81	0.70	0.56	0.70	-0.76	-0.42
PRIm3	-0.78	-0.67	0.56	0.70	-0.77	0.40
PRIm2	-0.77	-0.65	0.55	0.70	-0.77	0.37
RGI	-0.77	-0.65	0.55	0.71	-0.78	0.40
Cab	0.86	0.65	0.55	0.71	-0.77	-0.34
CRI550m	0.90	0.73	0.55	0.68	-0.75	-0.44
TcTa_InMn	-0.77	-0.52	0.54	0.68	-0.77	0.66
TcTa_InMed	-0.78	-0.50	0.54	0.68	-0.77	0.66
PSRI	-0.80	-0.56	0.53	0.70	-0.79	0.37
DRI_PRI	-0.81	-0.55	0.53	0.69	-0.78	0.33
DNCabxc	0.85	0.29	0.53	0.66	-0.76	-0.23
REP_Li2	-0.79	-0.56	0.53	0.68	-0.79	0.43
DNIRCabCxc	0.86	0.29	0.53	0.67	-0.76	-0.22
BF2	0.88	0.62	0.53	0.66	-0.77	-0.47
BF1	0.84	0.61	0.53	0.65	-0.77	-0.47
PRI	-0.02	-0.05	0.53	0.68	-0.78	-0.06
PRI528	-0.54	-0.36	0.53	0.72	-0.77	0.33
HI	0.74	0.50	0.53	0.70	-0.78	-0.35
BF3	0.88	0.48	0.53	0.66	-0.77	-0.32
REP_LE	0.85	0.41	0.53	0.68	-0.77	-0.31

The results are in order of decreasing mean RNIR_CRI700 EBV accuracy.

Modelling RNIR_CRI700 with the structure-intensive pigment index SIPI as the secondary trait increased its EBV accuracy by 6.8%, from 0.71 to 0.76. Modelling RNIR_CRI700 with the blue/green index BGI2 as the secondary trait increased its EBV accuracy by 4.3%, from 0.71 to 0.74. Using any other index as a secondary trait led to a decrease in EBV accuracy from the univariate analysis of RNIR_CRI700. The absolute correlation between RNIR_CRI700 EBVs and severity score EBVs ranged from 0.55 to 0.79. **Figure 6C** shows the relationship between RNIR_CRI700 EBVs from the bivariate analysis with SIPI, which has a Pearson correlation coefficient of -0.76 with severity scores. This correlation is less strong than the correlation calculated with RNIR_CRI700 EBVs from the univariate analysis (r_P_ = -0.79, **Figure 6B**), which suggests that jointly modeling traits adds complexity without necessarily improving the alignment of RNIR_CRI700 with severity scores. Using RNIR_CRI700 alone may therefore be more straightforward and equally effective.

## Discussion

This study leverages a Dothistroma needle blight outbreak displaying the full range of severity levels within a radiata pine clonal breeding trial to extend phenotyping methods for the assessment of disease susceptibility using remote sensing techniques. We quantified the extent of genetic control of novel thermal and spectral disease susceptibility phenotypes through a large set of replicated genotypes in a clonal breeding trial. Our research focused on identifying the most appropriate phenotypes and statistical methodologies for estimating genetic parameters and tested several operational scenarios for phenotyping. These scenarios included approaches solely based on remote sensing and those integrating remote sensing with traditional phenotyping techniques. Although a few studies have calculated narrow-sense heritability of spectral indices in tree species (Čepl et al., 2018; Tao et al., 2021), this is the first time that such indices have been used to characterise disease susceptibility in an operational breeding trial, and the first time field-based thermal measurements on forest stands have been used for tree genetic assessment. The successful combination of these measurement methods with UAV technology paves the way for a future implementation of high-throughput phenotyping of plant stress in forestry breeding trials.

The narrow-sense heritability values for visual severity scores obtained in this study align with previous estimates from breeding trials in New Zealand and Australia (Li et al., 2018; Ismael et al., 2020; Klápště et al., 2020). We confirmed that foliar disease modelling requires the addition of spatial structure through both trial-level geographical information (here trial rows and columns) and neighbourhood effects to take into account the landscape variability and mode of transmission of the disease. The application of single-step genomic evaluation, integrating pedigree and genomic marker information in the genetic relationship matrix, improved model performance. The optimal proportion of the ***G*** matrix, which reflects the weighting between pedigree and genomic marker information, was found to be between 0.4-0.6, which is consistent with previous studies within the same breeding programme (Klápště et al., 2020, 2022).

UAV hyperspectral and thermal data acquisition at the time of the outbreak enabled the remote phenotyping of disease severity through the calculation of spectral, thermal, and fluorescence indices. Pigment- and structure-sensitive narrow-band hyperspectral indices had a strong observed correlation with severity scores and a narrow-sense heritability similar to severity scores. The importance of these indices is consistent with previous research describing the impacts of DNB and disease on changes in tree structure and fundamental leaf-level physiological processes. Previous research has demonstrated the importance of carotenoids as a symptom of DNB severity (Watt et al., 2023b) and the carotenoid:chlorophyll ratio has been found to increase as a result of increasing disease severity (Watt et al., 2023b). This is consistent with our results of carotenoid-sensitive indices such as RNIR_CRI700, RNIR_CRI550, and PSSR_c showing highest correlation with severity scores. It was interesting to note that the RTM plant trait that performed best was carotenoid content (CAR). Although CAR did not perform as well as the pigment-related NBHIs discussed above, the relative importance of CAR amongst other RTM traits is consistent with rankings between indices. The utility of the important PRI indices (PRI_CI, PRI515, PRI550, PRIm1), which can account for xanthophyll dynamics, are consistent with a large body of previous research demonstrating their utility for detecting a range of stresses (Peguero-Pina et al., 2008; Zarco-Tejada et al., 2009; Watt et al., 2024a). Photochemical reflectance index is an effective proxy for the reductions in stomatal conductance and assimilation rate that are associated with pre-visual and early disease (Hernández-Clemente et al., 2019) and was found to be one of the most important classifiers of DNB severity within a different radiata pine field trial (Watt et al., 2023b). It is interesting to note that non-standard formulations of PRI appeared to be more important than the standard PRI which uses R_531_ as a xanthophyll-sensitive spectral band. Compared to the standard formulation, the PRI variants using bands from the 500–515 nm spectral region (i.e., PRI_m1_ and PRI_515_) have been shown to be less sensitive to leaf area index, tree densities, and structural effects in conifer canopies (Hernández-Clemente et al., 2011), which highlights again the importance of accounting for stand structure in identifying changes in pigments.

Canopy temperature indices from thermal captures showed promising performance in this study. Following energy balance theory, increases in leaf temperature result from reductions in stomatal conductance and lower rates of transpiration (Smith et al., 1986; Still et al., 2019), and our results show that these modifications are detectable by UAV thermal imagery at the canopy level.

Through an extensive simulation exercise, we showed that the joint modelling of severity scores and the best-performing NBHI, RNIR_CRI700, allows the estimation of severity scores breeding values with very high fidelity (absolute EBV correlation above 0.9) when at least 50% of the trial is manually scored. Even when only 20% of the trial is scored, the fidelity remains relatively high (absolute EBV correlation above 0.8). The associated loss in accuracy is minimal, only decreasing from 0.74 to 0.68 at 50% scoring and to 0.59 at 20% scoring. We found that using the remote sensing trait RNIR_CRI700 as the sole disease severity phenotype in a univariate analysis led to an EBV accuracy of 0.71 with an absolute correlation of 0.79 with severity scores. These results suggest that partial visual scoring could be entirely replaced by hyperspectral remote sensing, with little loss in EBV correlation and no reduction in EBV accuracy compared to a bivariate analysis that incorporates partial visual scoring. We also assessed the potential benefits of combining several indices to improve phenotyping from only one index. However, using a combination of remote sensing indices in a multi-trait setup did not improve the quality of genetic results over univariate results, as it only minorly increased EBV accuracy and considerably reduced the correlation of the selected remote sensing index EBVs with severity score EBVs. This can be explained by the redundancy of many of the pigment indices. Indeed, although we identified 21 promising pigment-related indices, we found a high level of correlation among them, which can partially be explained by the similarity in their calculation from spectral bands (**Supplementary Table S1**). We therefore suggest that using the remote sensing trait most correlated with traditional severity scores in a univariate analysis can be sufficient and leads to EBV estimations more in line with severity score EBVs than those estimated from the joint analysis of multiple remote sensing traits.

The focus of this study was restricted to exploring remote sensing options to supplement or replace currently time-consuming disease phenotyping methods, with the objective to maximise high fidelity of alternative methods to traditional ones. Although the technical requirements of high-quality spectral and thermal field captures presented here might currently be prohibitive in the forestry context, we found that many of the high-heritability indices use spectral bands that can be captured with multispectral instruments, which are more accessible than hyperspectral ones. As UAV remote-sensing assessments become more routine and gain trust among foresters and tree breeders, we believe the advantages of remote sensing for tree disease phenotyping will expand and outweigh traditional methods. First, the precision, accuracy, and consistency of remote-sensing measurements may lead to more effective breeding outcomes than with traditional phenotyping. Also, structural traits captured in years following disease using LiDAR or spectral methods or derived through radiative transfer modelling such as leaf area index can complement hyperspectral and thermal data to assess longer-term outcomes of disease on tree health such as defoliation. As a number of stress responses detected by hyperspectral and thermal metrics are likely common to several different diseases, remote sensing phenotyping of stress responses might be useful in simultaneously selecting for resistance to multiple foliar diseases. Ismael et al. (2020) showed evidence for strong genetic correlation of resistance to multiple foliar diseases such as Dothistroma needle blight and Cyclaneusma needle cast in radiata pine. More generally, hyperspectral and thermal indices can be used not only for disease-related phenotypes but also for other biotic and non-biotic stress responses. For instance, canopy temperature can specifically be used to characterise drought stress phenotypes, as it is a proxy for stomatal conductance, transpiration and assimilation rate.

## Conclusions

Our study illustrates the potential for the remote sensing capture of hyperspectral and thermal traits to be used in disease resistance phenotyping for tree breeding. Genotype performance for traditional and remote sensing phenotypes was enhanced by the use of genomic and pedigree data through spatially explicit single-step predictive modelling and led to high breeding value accuracy. Single narrow-band hyperspectral indices calculated on all trees in the breeding trial can be used to complement partial visual disease scoring or replace it altogether with relatively high fidelity, with close to no loss in breeding value accuracy. However, the success of high-throughput single-tree characterisation of hyperspectral and thermal traits in a field trial setup achieved in this study is highly innovative and required a large amount of expertise and resources. Additional advanced experimentation in field methodologies will help streamline and reduce the cost of data capture processes. This will enable the extension of this disease phenotyping approach to other trials and other phenotyping applications and solidify the adequate identification of remote sensing metrics as phenotypes for physiological traits of interest.

## Data Availability

The datasets generated for this study can be found in the Dryad data repository: DOI: 10.5061/dryad.5hqbzkhhk.
