# Supplementary material for: Leveraging UAV spectral and thermal traits for the genetic improvement of resistance to Dothistroma needle blight in Pinus radiata D.Don
Source: Front Plant Sci. 2025 Jun 17;16:1574720. doi: 10.3389/fpls.2025.1574720 (PMC12209191; doi:10.3389/fpls.2025.1574720)
Supplement: Supplementary file 1 [file Table1.pdf]

Table S1. Narrow-band hyperspectral indices (NBHI), radiative transfer model inverted (RTM) traits, solar-induced fluorescence indices, and thermal indices used in the analysis, with results from genetic analyses. The indices with estimated heritability values above 0.3 are in bold. Mult.comp.: Compatibility of index calculation with multispectral capture; h^2: estimated narrow-sense heritability; Obs. cor.: observed correlation; EBV cor.: correlation of estimated breeding values (EBVs) with EBVs from severity scores.

Index name	Index code	Equation	Mult. comp.	Obs. cor. with severity	h^2	EBV cor.	Mean EBV accuracy	Reference
**Structural indices**								
Chlorophyll Abs. Reflectance Index	CARI	$(R_{700} - R_{670}) - 0.2(R_{700} - R_{550})$	yes	0.005	0.094	-0.209	0.543	Kim (1994)
Enhanced Vegetation Index	EVI	$2.5 \cdot (R_{800} - R_{670})/(R_{800} + 6 \cdot R_{670} - 7.5 \cdot R_{800} + 1)$	yes	0.004	0.000	-0.024	0.352	Liu and Huete (1995)
Modified Chlorophyll Abs. Index	MCARI	$[(R_{700} - R_{670}) - 0.2(R_{700} - R_{550})] \cdot (R_{700}/R_{670})$	yes	-0.256	0.148	-0.460	0.618	Daughtry et al. (2000)
**Modified Chlorophyll Abs. Index 3**	**MCARI3**	$[(R_{750} - R_{705}) - 0.2(R_{750} - R_{550})] \cdot (R_{750}/R_{705})$	yes	-0.737	**0.335**	-0.786	0.694	Wu et al. (2008)
Modified Soil-Adjusted Vegetation Index	MSAVI	$\frac{2 \cdot R_{800} + 1 - \sqrt{(2 \cdot R_{800} + 1)^2 - 8(R_{800} - R_{670})}}{2}$	yes	-0.667	0.255	-0.768	0.672	Qi et al. (1994)
**Modified Simple Ratio**	**MSR**	$\frac{R_{800}/R_{670} - 1}{(R_{800}/R_{670})^{0.5} + 1}$	yes	-0.755	**0.324**	-0.794	0.693	Chen (1996)
Modified Triangular Veg. Index 1	MTVI1	$1.2[1.2(R_{800} - R_{550}) - 2.5(R_{670} - R_{550})]$	yes	-0.603	0.218	-0.738	0.655	Haboudane et al. (2004)
Modified Triangular Veg. Index 2	MTVI2	$\frac{1.5[1.2(R_{800} - R_{550}) - 2.5(R_{670} - R_{550})]}{\sqrt{(2R_{800} + 1)^2 - (6R_{800} - 5\sqrt{R_{670}}) - 0.5}}$	yes	-0.691	0.274	-0.780	0.679	Haboudane et al. (2004)
Denominator of MTVI2	MTVI2_den	$\sqrt{(2R_{800} + 1)^2 - (6R_{800} - 5\sqrt{R_{670}}) - 0.5}$	yes	0.636	0.175	0.678	0.613	Haboudane et al. (2004)
Normalized Difference Veg. Index	NDVI	$(R_{800} - R_{670})/(R_{800} + R_{670})$	yes	-0.745	0.266	-0.783	0.677	Rouse et al. (1974)
NDVI using R750 & R705	NDVI2	$(R_{750} - R_{705})/(R_{750} + R_{705})$	yes	-0.766	0.281	-0.789	0.680	Gitelson and Merzlyak (1994a)
Optimized Soil-Adjusted Veg. Index	OSAVI	$((1 + 0.16) \cdot (R_{800} - R_{670})/(R_{800} + R_{670} + 0.16))$	yes	-0.720	0.270	-0.787	0.679	Rondeaux et al. (1996)

Renormalized Difference Veg. Index	RDVI	$(R_{800} - R_{670})/\sqrt{(R_{800} + R_{670})}$	yes	-0.690	0.258	-0.776	0.673	Roujean and Breon (1995)
**Simple Ratio**	**SR**	R_{800}/R_{670}	yes	-0.734	**0.333**	-0.779	0.692	Jordan (1969)
Triangular Vegetation Index	TVI	$0.5 \cdot [120 \cdot (R_{750} - R_{550}) - 200 \cdot (R_{670} - R_{550})]$	yes	-0.588	0.212	-0.729	0.652	Broge and Leblanc (2001)
**<u>Pigment indices</u>**								
(Anth.) Reflectance index (AnthRI)	antARI	$\left[{R_{530}}^{-1} - (R_{670-676})^{-1}\right] \cdot R_{772-880}$		-0.078	0.117	0.068	0.604	Matsuda et al. (2012)
Anthocyanins Index	ARI	${R_{550}}^{-1} - {R_{700}}^{-1}$	yes	0.136	0.117	0.363	0.604	Gitelson et al. (2001)
Anthocyanins Reflectance index 2	ARI2	${R_{559}}^{-1} - {R_{721}}^{-1}$	yes	-0.517	0.136	-0.478	0.549	Gitelson et al. (2001)
Carter indices	CAR	R_{695}/R_{760}		0.734	0.251	0.775	0.672	Carter et al. (1996)
Carter indices	CTR1	R_{695}/R_{420}		-0.160	0.170	-0.179	0.657	Carter (1994)
**Chlorophyll Index Red Edge**	**CI**	R_{750}/R_{710}	yes	-0.769	**0.317**	-0.784	0.691	Zarco-Tejada et al. (2001)
Carotenoid Reflectance Indices	CRI550	$(1/R_{510}) - (1/R_{550})$		-0.704	0.254	-0.738	0.665	Gitelson et al. (2006); Gitelson et al. (2003)
Carotenoid Reflectance Indices	CRI550_515	$(1/R_{515}) - (1/R_{550})$		-0.700	0.244	-0.737	0.657	Gitelson et al. (2006)
Carotenoid Reflectance Indices	CRI700	$(1/R_{510}) - (1/R_{700})$		-0.626	0.186	-0.636	0.615	Gitelson et al. (2006); Gitelson et al. (2003)
Carotenoid Reflectance Indices	CRI700_515	$(1/R_{515}) - (1/R_{700})$		-0.585	0.159	-0.583	0.586	Gitelson et al. (2006)
Reflectance band ratio indices	DCabCxc	$R_{672}/(R_{550} \cdot 3R_{708})$		0.215	0.121	0.443	0.607	Datt (1998)
Reflectance band ratio indices	DNIRCabCxc	$R_{860}/(R_{550} \cdot R_{708})$	yes	-0.664	0.221	-0.655	0.634	Datt (1998)
**Gitelson & Merzlyak index 1**	**GM1**	R_{750}/R_{550}	yes	-0.791	**0.328**	-0.784	0.694	Gitelson and Merzlyak (1997)
**Gitelson & Merzlyak index 2**	**GM2**	R_{750}/R_{700}	yes	-0.755	**0.332**	-0.787	0.694	Gitelson and Merzlyak (1997)
**Gitelson & Merzlyak index 4**	**GM4**	R_{750}/R_{555}	yes	-0.790	**0.329**	-0.783	0.694	Gitelson and Merzlyak (1994b)

Modified anthocyanins Reflectance index (mARI)	MARI	$(R_{550}{}^{-1} - R_{770}{}^{-1}) \cdot R_{780}$		-0.254	0.141	-0.078	0.622	Rahimzadeh-Bajgiran et al. (2012)
Normalized Pigments Index	NPCI	$(R_{680} - R_{430})/(R_{680} + R_{430})$		0.127	0.131	0.165	0.620	Penuelas et al. (1995)
Normalized Phaeophytinization Index	NPQI	$(R_{415} - R_{435})/(R_{415} + R_{435})$	yes	-0.185	0.157	-0.011	0.652	Penuelas et al. (1995)
Pigment Specific Normalized Difference	PSNDc	$(R_{800} - R_{470})/(R_{800} + R_{470})$		-0.786	0.292	-0.801	0.684	Blackburn (1998)
Plant Senescencing Reflectance Index	PSRI	$(R_{680} - R_{500})/R_{750}$		0.650	0.216	0.702	0.662	Merzlyak et al. (1999)
**Pigment Specific Simple Ratio Chlorophyll a**	**PSSR_a**	R_{800}/R_{675}		-0.732	**0.329**	-0.778	0.690	Blackburn (1998)
**Pigment Specific Simple Ratio Chlorophyll b**	**PSSR_b**	R_{800}/R_{650}		-0.748	**0.342**	-0.785	0.697	Blackburn (1998)
**Pigment Specific Simple Ratio Carotenoids**	**PSSR_c**	R_{800}/R_{500}		-0.786	**0.365**	-0.800	0.708	Blackburn (1998)
**Carotenoid Reflectance Index**	**RNIR_CRI550**	$(1/R_{510}) - (1/R_{550}) \cdot R_{770}$		-0.777	**0.372**	-0.797	0.711	Gitelson et al. (2006); Gitelson et al. (2003)
**Carotenoid Reflectance Index**	**RNIR_CRI700**	$(1/R_{510}) - (1/R_{700}) \cdot R_{770}$		-0.793	**0.371**	-0.792	0.712	Gitelson et al. (2006); Gitelson et al. (2003)
Reciprocal reflectance	RR	$1/R_{700}$	yes	-0.568	0.156	-0.596	0.577	Gitelson et al. (1999)
Structure-Intensive Pigment Index	SIPI	$(R_{800} - R_{445})/(R_{800} + R_{680})$	yes	0.645	0.202	0.690	0.651	Penuelas et al. (1995)
Simple Ratio Pigment Index	SRPI	R_{430}/R_{680}		-0.127	0.127	-0.165	0.615	Penuelas et al. (1995)
Transformed Chlorophyll Absorption in Reflectance Index	TCARI	$3 \cdot [(R_{700} - R_{670}) - 0.2 \cdot (R_{700} - R_{550}) \cdot (R_{700}/R_{670})$	yes	-0.068	0.098	-0.295	0.559	Haboudane et al. (2002)
Transformed Chlorophyll Absorption in Reflectance Index/ Optimized Soil-Adjusted Vegetation Index	TCARI_OSAVI	$\frac{3 \cdot [(R_{700} - R_{670}) - 0.2 \cdot (R_{700} - R_{550}) \cdot (R_{700}/R_{670})}{((1 + 0.16) \cdot (R_{800} - R_{670})/(R_{800} + R_{670} + 0.16))}$	yes	0.405	0.123	0.302	0.534	Haboudane et al. (2002)
Vogelmann indices	VOG1	R_{740}/R_{720}		-0.771	0.297	-0.775	0.682	Vogelmann (1993)
Vogelmann indices	VOG2	$(R_{734} - R_{747})/(R_{715} + R_{726})$		0.761	0.296	0.760	0.682	Vogelmann (1993)
**Vogelmann indices**	**VOG3**	$(R_{734} - R_{747})/(R_{715} + R_{720})$		0.762	**0.302**	0.761	0.684	Vogelmann (1993)

**Chlorophyll indices**								
Red-edge inflection point	REP_Deriv1	See reference		-0.227	0.005	-0.277	0.372	Horler et al. (1983)
Red-edge inflection point	REP_Deriv2	See reference		-0.006	0.000	0.177	0.351	Horler et al. (1983)
Red-edge position linear extrapolation	REP_LE	See reference		-0.709	0.244	-0.667	0.653	Cho and Skidmore (2006)
Red-edge position linear interpolation	REP_Li2	$700 + 40 \cdot (R_{670} + R_{780}/2)/(R_{740} - R_{700})$		0.674	0.211	0.724	0.654	Cho and Skidmore (2006)
Sum of first derivative reflectance between *R*680 and *R*780	Sum_Dr2	See reference		-0.503	0.171	-0.673	0.633	Filella and Penuelas (1994)
Reflectance Curvature Index	CUR	$(R_{675} \cdot R_{690})/R_{683}^2$		-0.548	0.184	-0.633	0.650	Zarco-Tejada et al. (2000)
**Xanthophyll indices**								
**Carotenoid/Chlorophyll Ratio Index**	**PRI_CI**	$(R_{570} - R_{530})/(R_{570} + R_{530}) \cdot ((R_{760}/R_{700}) - 1)$		-0.758	**0.317**	-0.804	0.693	Garrity et al. (2011)
**Photochemical Refl. Index (515)**	**PRI515**	$(R_{515} - R_{531})/(R_{515} + R_{531})$		0.736	**0.343**	0.775	0.709	Hernández-Clemente et al. (2011)
Photochemical Refl. Index (528)	PRI528	$(R_{528} - R_{531})/(R_{528} + R_{531})$	yes	0.518	0.286	0.523	0.705	Gamon et al. (1992)
**Photochemical Refl. Index (550)**	**PRI550**	$(R_{550} - R_{531})/(R_{550} + R_{531})$	yes	-0.747	**0.342**	-0.764	0.712	Gamon et al. (1992)
Photochemical Refl. Index (570)	PRI570	$(R_{570} - R_{531})/(R_{570} + R_{531})$	yes	0.064	0.183	0.107	0.661	(Gamon et al., 1992)
**Photochemical Refl. Index (512)**	**PRIm1**	$(R_{512} - R_{531})/(R_{512} + R_{531})$		0.734	**0.343**	0.773	0.709	Hernández-Clemente et al. (2011)
Photochemical Refl. Index (600)	PRIm2	$(R_{600} - R_{531})/(R_{600} + R_{531})$		0.582	0.244	0.673	0.676	(Gamon et al., 1992)
Photochemical Refl. Index (670)	PRIm3	$(R_{670} - R_{531})/(R_{670} + R_{531})$	yes	0.598	0.237	0.694	0.671	(Gamon et al., 1992)
Photochemical Refl. Index (670 and 570)	PRIm4	$(R_{570} - R_{531} - R_{670})/(R_{570} + R_{531} + R_{670})$	yes	-0.647	0.267	-0.727	0.685	Hernández-Clemente et al. (2011)

Normalized Photoch. Refl. Index	PRIn	$PRI_{570}/[RDVI \cdot (R_{700}/R_{670})]$	yes	0.552	0.196	0.652	0.653	Zarco-Tejada et al. (2013)
**R/G/B indices**								
Blue Index	B	R_{450}/R_{490}	yes	0.221	0.190	0.260	0.650	Calderón et al. (2013)
BF1	BF1	R_{400}/R_{410}		-0.683	0.215	-0.676	0.643	Zarco-Tejada et al. (2018)
BF2	BF2	R_{400}/R_{420}	yes	-0.705	0.226	-0.708	0.642	Zarco-Tejada et al. (2018)
BF3	BF3	R_{400}/R_{430}		-0.691	0.206	-0.653	0.632	Zarco-Tejada et al. (2018)
BF4	BF4	R_{400}/R_{440}		-0.648	0.182	-0.607	0.624	Zarco-Tejada et al. (2018)
BF5	BF5	R_{400}/R_{450}		-0.615	0.164	-0.556	0.616	Zarco-Tejada et al. (2018)
Blue/green index	BGI1	R_{400}/R_{550}		0.168	0.170	0.405	0.647	Zarco-Tejada et al. (2005)
**Blue/green index**	**BGI2**	R_{450}/R_{550}	yes	0.766	**0.370**	0.784	0.723	Zarco-Tejada et al. (2005)
Blue/red indices	BRI1	R_{400}/R_{690}		-0.524	0.147	-0.508	0.612	Zarco-Tejada et al. (2012)
Blue/red indices	BRI2	R_{450}/R_{690}	yes	-0.167	0.152	-0.202	0.634	Zarco-Tejada et al. (2012)
Greenness Index	G	R_{570}/R_{670}	yes	-0.628	0.261	-0.716	0.674	Calderón et al. (2013)
Greenness Index 2	GI	R_{554}/R_{677}	yes	-0.630	0.261	-0.721	0.673	Zarco-Tejada et al. (2005)
Lichtenthaler Index 1	LIC1	$(R_{800} - R_{680})/(R_{800} + R_{680})$	yes	-0.741	0.262	-0.781	0.676	Lichtenthaler (1996)
Lichtenthaler Index 2	LIC2	R_{440}/R_{690}	yes	-0.079	0.140	-0.102	0.627	Lichtenthaler (1996)
**Lichtenthaler Index 3**	**LIC3**	R_{440}/R_{740}	yes	0.790	**0.304**	0.806	0.690	Lichtenthaler (1996)
Redness Index	R	R_{700}/R_{670}	yes	-0.668	0.298	-0.733	0.689	Gitelson et al. (2000)
**Ratio Analysis of Reflectance Spectra**	**RARS**	R_{746}/R_{513}	yes	-0.791	**0.356**	-0.805	0.705	Chappelle et al. (1992)
Red/green indices	RGI	R_{690}/R_{550}		0.604	0.235	0.701	0.676	Zarco-Tejada et al. (2005)
**Water indices**								
Floating position Water Band index	fWBI	$R_{900}/min_{R930-R980}$		-0.366	0.044	-0.362	0.448	Strachan et al. (2002)
Water Band Index using R950 & R900	SR_WBI	R_{950}/R_{900}		0.065	0.004	-0.049	0.372	Penuelas et al. (1993)
Water Band Index	WBI	R_{970}/R_{900}		0.375	0.040	0.378	0.437	Peñuelas et al. (1993)
Water Index	WI	R_{900}/R_{970}		-0.362	0.039	-0.362	0.437	Peñuelas et al. (1997)

**Plant disease indices**								
Cercospora leaf spot index	CLS	$\frac{R_{698} - R_{570}}{R_{698} + R_{570}} - R_{734}$	yes	0.516	0.203	0.680	0.655	Mahlein et al. (2013)
Healthy-index	HI	$\frac{R_{534} - R_{698}}{R_{534} + R_{698}} - \frac{1}{2} \cdot R_{704}$		-0.571	0.222	-0.658	0.665	Mahlein et al. (2013)
Powdery mildew index	PMI	$\frac{R_{520} - R_{584}}{R_{520} + R_{584}} + R_{724}$		0.100	0.053	-0.047	0.510	Mahlein et al. (2013)
**Sugar beet rust–index**	**SBRI**	$\frac{R_{570} - R_{513}}{R_{570} + R_{513}} + \frac{1}{2} \cdot R_{704}$		-0.697	**0.363**	-0.709	0.722	Mahlein et al. (2013)
**RTM inverted traits**								
Anthocyanin content	Ant	-		0.226	0.132	0.418	0.619	Poblete et al. (submitted)
Chlorophyll content	Cab	-		-0.710	0.281	-0.695	0.677	Poblete et al. (submitted)
Carotenoid content	Car_RTM	-		-0.692	0.254	-0.758	0.666	Poblete et al. (submitted)
Leaf area index	LAI	-		-0.614	0.176	-0.672	0.599	Poblete et al. (submitted)
Average leaf angle	LIDFa	-		0.500	0.165	0.676	0.626	Poblete et al. (submitted)
**Solar-induced fluorescence**								
Solar-induced fluorescence	2FLD_resampled	-		-0.283	0.057	-0.534	0.523	Mohammed et al. (2019)
Solar-induced fluorescence	3FLD_resampled	-		-0.156	0.017	-0.415	0.439	Maier et al. (2004)
**Thermal indices**								
Median normalised canopy temperature	TcTa_InMed	-		0.752	0.215	0.787	0.679	Idso et al. (1981)
Mean normalised canopy temperature	TcTa_InMn	-		0.742	0.210	0.776	0.680	Idso et al. (1981)
Standard deviation of the normalised canopy temperature	TcTa_InStdev	-		-0.123	0.128	-0.354	0.607	Idso et al. (1981)

References

Blackburn, G.A. (1998). Spectral indices for estimating photosynthetic pigment concentrations: a test using senescent tree leaves. *International Journal of remote sensing* 19(4), 657-675.

Broge, N.H., and Leblanc, E. (2001). Comparing prediction power and stability of broadband and hyperspectral vegetation indices for estimation of green leaf area index and canopy chlorophyll density. *Remote sensing of environment* 76(2), 156-172.

Calderón, R., Navas-Cortés, J.A., Lucena, C., and Zarco-Tejada, P.J. (2013). High-resolution airborne hyperspectral and thermal imagery for early detection of Verticillium wilt of olive using fluorescence, temperature and narrow-band spectral indices. *Remote Sensing of Environment* 139, 231-245.

Carter, G.A. (1994). Ratios of leaf reflectances in narrow wavebands as indicators of plant stress. *Remote Sensing* 15(3), 697-703.

Carter, G.A., Cibula, W.G., and Dell, T.R. (1996). Spectral reflectance characteristics and digital imagery of a pine needle blight in the southeastern United States. *Canadian Journal of Forest Research* 26(3), 402-407.

Chappelle, E.W., Kim, M.S., and Mcmurtrey, J.E. (1992). Ratio analysis of reflectance spectra (RARS): An algorithm for the remote estimation of the concentrations of Chlorophyll A, Chlorophyll B, and Carotenoids in Soybean leaves. *Remote Sensing of Environment* 39, 239-247.

Chen, J.M. (1996). Evaluation of vegetation indices and a modified simple ratio for boreal applications. *Canadian Journal of Remote Sensing* 22(3), 229-242.

Cho, M.A., and Skidmore, A.K. (2006). A new technique for extracting the red edge position from hyperspectral data: The linear extrapolation method. *Remote sensing of environment* 101(2), 181-193.

Datt, B. (1998). Remote sensing of chlorophyll a, chlorophyll b, chlorophyll a+ b, and total carotenoid content in eucalyptus leaves. *Remote Sensing of Environment* 66(2), 111-121.

Daughtry, C.S.T., Walthall, C.L., Kim, M.S., De Colstoun, E.B., and McMurtrey Iii, J.E. (2000). Estimating corn leaf chlorophyll concentration from leaf and canopy reflectance. *Remote sensing of Environment* 74(2), 229-239.

Filella, I., and Penuelas, J. (1994). The red edge position and shape as indicators of plant chlorophyll content, biomass and hydric status. *International journal of remote sensing* 15(7), 1459-1470.

Gamon, J.A., Penuelas, J., and Field, C.B. (1992). A narrow-waveband spectral index that tracks diurnal changes in photosynthetic efficiency. *Remote Sensing of environment* 41(1), 35-44.

Garrity, S.R., Eitel, J.U.H., and Vierling, L.A. (2011). Disentangling the relationships between plant pigments and the photochemical reflectance index reveals a new approach for remote estimation of carotenoid content. *Remote Sensing of Environment* 115(2), 628-635.

Gitelson, A., and Merzlyak, M.N. (1994a). Quantitative estimation of chlorophyll-a using reflectance spectra: Experiments with autumn chestnut and maple leaves. *Journal of Photochemistry and Photobiology B: Biology* 22(3), 247-252.

Gitelson, A.A., Buschmann, C., and Lichtenthaler, H.K. (1999). The chlorophyll fluorescence ratio F735/F700 as an accurate measure of the chlorophyll content in plants. *Remote sensing of environment* 69(3), 296-302.

Gitelson, A.A., Gritz, Y., and Merzlyak, M.N. (2003). Relationships between leaf chlorophyll content and spectral reflectance and algorithms for non-destructive chlorophyll assessment in higher plant leaves. *Journal of plant physiology* 160(3), 271-282.

Gitelson, A.A., Keydan, G.P., and Merzlyak, M.N. (2006). Three-band model for noninvasive estimation of chlorophyll, carotenoids, and anthocyanin contents in higher plant leaves. *Geophysical research letters* 33(11).

Gitelson, A.A., and Merzlyak, M.N. (1994b). Spectral reflectance changes associated with autumn senescence of Aesculus hippocastanum L. and Acer platanoides L. leaves. Spectral features and relation to chlorophyll estimation. *Journal of plant physiology* 143(3), 286-292.

Gitelson, A.A., and Merzlyak, M.N. (1997). Remote estimation of chlorophyll content in higher plant leaves. *International Journal of Remote Sensing* 18(12), 2691-2697.

Gitelson, A.A., Merzlyak, M.N., and Chivkunova, O.B. (2001). Optical properties and nondestructive estimation of anthocyanin content in plant leaves¶. *Photochemistry and photobiology* 74(1), 38-45.

Gitelson, A.A., Yacobi, Y.Z., Schalles, J.F., Rundquist, D.C., Han, L., Stark, R., et al. (2000). Remote estimation of phytoplankton density in productive waters. *Advances in limnology. Stuttgart* 55**,** 121-136.

Haboudane, D., Miller, J.R., Pattey, E., Zarco-Tejada, P.J., and Strachan, I.B. (2004). Hyperspectral vegetation indices and novel algorithms for predicting green LAI of crop canopies: Modeling and validation in the context of precision agriculture. *Remote sensing of environment* 90(3)**,** 337-352.

Haboudane, D., Miller, J.R., Tremblay, N., Zarco-Tejada, P.J., and Dextraze, L. (2002). Integrated narrow-band vegetation indices for prediction of crop chlorophyll content for application to precision agriculture. *Remote sensing of environment* 81(2-3)**,** 416-426.

Hernández-Clemente, R., Navarro-Cerrillo, R.M., Suárez, L., Morales, F., and Zarco-Tejada, P.J. (2011). Assessing structural effects on PRI for stress detection in conifer forests. *Remote Sensing of Environment* 115(9)**,** 2360-2375.

Horler, D., Dockray, M., and Barber, J. (1983). The red edge of plant leaf reflectance. *International journal of remote sensing* 4(2)**,** 273-288.

Idso, S.B., Jackson, R.D., Pinter Jr, P.J., Reginato, R.J., and Hatfield, J.L. (1981). Normalizing the stress-degree-day parameter for environmental variability. *Agricultural Meteorology* 24**,** 45-55.

Jordan, C.F. (1969). Derivation of leaf-area index from quality of light on the forest floor. *Ecology* 50(4)**,** 663-666.

Kim, M.S. (1994). The use of narrow spectral bands for improving remote sensing estimations of fractionally absorbed photosynthetically active radiation. Ph.D. Dissertation, University of Maryland, College Park, MD, USA.

Lichtenthaler, H.K. (1996). Vegetation stress: an introduction to the stress concept in plants. *Journal of plant physiology* 148(1-2)**,** 4-14.

Liu, H.Q., and Huete, A. (1995). A feedback based modification of the NDVI to minimize canopy background and atmospheric noise. *IEEE Transactions on Geoscience and Remote Sensing* 33(2)**,** 457-465.

Mahlein, A.K., Rumpf, T., Welke, P., Dehne, H.W., Plümer, L., Steiner, U., et al. (2013). Development of spectral indices for detecting and identifying plant diseases. *Remote Sensing of Environment* 128**,** 21-30. doi: 10.1016/j.rse.2012.09.019.

Maier, S.W., Günther, K.P., and Stellmes, M. (2004). Sun-induced fluorescence: A new tool for precision farming. *Digital imaging and spectral techniques: Applications to precision agriculture and crop physiology* 66**,** 207-222.

Matsuda, O., Tanaka, A., Fujita, T., and Iba, K. (2012). Hyperspectral imaging techniques for rapid identification of Arabidopsis mutants with altered leaf pigment status. *Plant and Cell Physiology* 53(6)**,** 1154-1170.

Merzlyak, M.N., Gitelson, A.A., Chivkunova, O.B., and Rakitin, V.Y.U. (1999). Non-destructive optical detection of pigment changes during leaf senescence and fruit ripening. *Physiologia plantarum* 106(1)**,** 135-141.

Mohammed, G.H., Colombo, R., Middleton, E.M., Rascher, U., van der Tol, C., Nedbal, L., et al. (2019). Remote sensing of solar-induced chlorophyll fluorescence (SIF) in vegetation: 50 years of progress. *Remote sensing of environment* 231**,** 111177.

Penuelas, J., Baret, F., and Filella, I. (1995). Semi-empirical indices to assess carotenoids/chlorophyll a ratio from leaf spectral reflectance. *Photosynthetica* 31(2)**,** 221-230.

Peñuelas, J., Filella, I., Biel, C., Serrano, L., and Save, R. (1993). The reflectance at the 950–970 nm region as an indicator of plant water status. *International journal of remote sensing* 14(10)**,** 1887-1905.

Penuelas, J., Gamon, J.A., Griffin, K.L., and Field, C.B. (1993). Assessing community type, plant biomass, pigment composition, and photosynthetic efficiency of aquatic vegetation from spectral reflectance. *Remote Sensing of Environment* 46(2)**,** 110-118.

Peñuelas, J., Pinol, J., Ogaya, R., and Filella, I. (1997). Estimation of plant water concentration by the reflectance water index WI (R900/R970). *International journal of remote sensing* 18(13)**,** 2869-2875.

Poblete, T., Watt, M.S., Main, R., Hartley, R., Estarija, H.C., McMillan, A.M.S., et al. (submitted). Early detection and characterisation of dothistroma needle blight on radiata pine using SIF, hyperspectral traits and canopy temperature. *Remote Sensing of Environment*.

Qi, J., Chehbouni, A., Huete, A.R., Kerr, Y.H., and Sorooshian, S. (1994). A modified soil adjusted vegetation index. *Remote sensing of environment* 48(2)**,** 119-126.

Rahimzadeh-Bajgiran, P., Munehiro, M., and Omasa, K. (2012). Relationships between the photochemical reflectance index (PRI) and chlorophyll fluorescence parameters and plant pigment indices at different leaf growth stages. *Photosynthesis research* 113**,** 261-271.

Rondeaux, G., Steven, M., and Baret, F. (1996). Optimization of soil-adjusted vegetation indices. *Remote sensing of environment* 55(2), 95-107.
Roujean, J.-L., and Breon, F.-M. (1995). Estimating PAR absorbed by vegetation from bidirectional reflectance measurements. *Remote sensing of Environment* 51(3), 375-384.
Rouse, J.W., Haas, R.H., Schell, J.A., and Deering, D.W. (1974). Monitoring vegetation systems in the Great Plains with ERTS. *NASA Special Publication* 351(1974), 309.
Strachan, I.B., Pattey, E., and Boisvert, J.B. (2002). Impact of nitrogen and environmental conditions on corn as detected by hyperspectral reflectance. *Remote Sensing of environment* 80(2), 213-224.
Vogelmann, T.C. (1993). Plant tissue optics. *Annual review of plant biology* 44(1), 231-251.
Wu, C., Niu, Z., Tang, Q., and Huang, W. (2008). Estimating chlorophyll content from hyperspectral vegetation indices: Modeling and validation. *Agricultural and Forest Meteorology* 148(8-9), 1230-1241.
Zarco-Tejada, P.J., Berjón, A., Lopez-Lozano, R., Miller, J.R., Martín, P., Cachorro, V., et al. (2005). Assessing vineyard condition with hyperspectral indices: Leaf and canopy reflectance simulation in a row-structured discontinuous canopy. *Remote Sensing of Environment* 99(3), 271-287.
Zarco-Tejada, P.J., Camino, C., Beck, P.S.A., Calderon, R., Hornero, A., Hernández-Clemente, R., et al. (2018). Previsual symptoms of Xylella fastidiosa infection revealed in spectral plant-trait alterations. *Nat. Plants* 4(7), 432-439.
Zarco-Tejada, P.J., González-Dugo, V., and Berni, J.A.J. (2012). Fluorescence, temperature and narrow-band indices acquired from a UAV platform for water stress detection using a micro-hyperspectral imager and a thermal camera. *Remote sensing of environment* 117, 322-337.
Zarco-Tejada, P.J., Miller, J.R., Mohammed, G.H., and Noland, T.L. (2000). Chlorophyll fluorescence effects on vegetation apparent reflectance: I. Leaf-level measurements and model simulation. *Remote Sensing of Environment* 74(3), 582-595.
Zarco-Tejada, P.J., Miller, J.R., Noland, T.L., Mohammed, G.H., and Sampson, P.H. (2001). Scaling-up and model inversion methods with narrowband optical indices for chlorophyll content estimation in closed forest canopies with hyperspectral data. *IEEE Transactions on Geoscience and Remote Sensing* 39(7), 1491-1507.
Zarco-Tejada, P.J., Morales, A., Testi, L., and Villalobos, F.J. (2013). Spatio-temporal patterns of chlorophyll fluorescence and physiological and structural indices acquired from hyperspectral imagery as compared with carbon fluxes measured with eddy covariance. *Remote Sensing of Environment* 133, 102-115.
