# Supplementary material for: Leveraging UAV spectral and thermal traits for the genetic improvement of resistance to Dothistroma needle blight in Pinus radiata D.Don
Source: Front Plant Sci. 2025 Jun 17;16:1574720. doi: 10.3389/fpls.2025.1574720 (PMC12209191; doi:10.3389/fpls.2025.1574720)
Supplement: Supplementary file 2 [file Table2.pdf]

Table S2. Ranges of parameters used to perform simulations with the PRO4SAIL2 radiative transfer model.

| **Parameter** | **Abbreviation** | **Units** | **Value / Range** |
|---|---|---|---|
| Chlorophyll content | $C_{a+b}$ | [µg/cm²] | [4,70] |
| Carotenoid content | $C_{x+c}$ | [µg/cm²] | [1,15] |
| Anthocyanin content | Anth. | [µg/cm²] | [1,12] |
| Dry matter content | $C_m$ | [g/cm²] | [0.005,0.04] |
| Water content | $C_w$ | [g/cm²] | [0.004,0.08] |
| Mesophyll struct. coeff. | N | - | [1.2,2.2] |
| Leaf area index | LAI | [m²/m²] | [1,7] |
| Average leaf angle | $LIDF_a$ | [deg.] | [1,90]; spherical |
| Hot spot parameter | hot | - | 0.01 |
| Observer angle | tto | [deg.] | 0 |
| Sun zenith angle | tts | [deg.] | 45 |
| Relative azimuth angle | psi | [deg.] | 0 |
| *Clumping parameters* | | | |
| Vertical crown cover | $C_v$ | - | [0-0.1] |
| Fraction of brown pigments | $F_b$ | - | 0 |
| Tree shape factor | Zeta | - | [0.2-0.6] |
| Dissociation factor | D | - | [0.8-1] |
